# Threshold Photoelectron Spectroscopy, Dissociative Photoionization, and Pyrolysis of Aziridine

**DOI:** 10.1021/acs.jpca.5c08612

**Published:** 2026-06-15

**Authors:** Henry Cardwell, Domenik Schleier, Jens Petersen, Michael Wenzel, Rory McClish, Jerry Kamer, Patrick Hemberger, Andras Bodi, Roland Mitrić, Jordy Bouwman

**Affiliations:** † Laboratory for Atmospheric and Space Physics, University of Colorado, Boulder, Colorado 80303, United States; ‡ Department of Chemistry, University of Colorado, Boulder, Colorado 80309, United States; § Institut für Physik und Astronomie, Technische Universität Berlin, Hardenbergstr. 36, Berlin 10623, Germany; ∥ University of Würzburg, Institute of Physical and Theoretical Chemistry, Am Hubland 97074 Würzburg, Germany; ⊥ Laboratory for Astrophysics, Leiden Observatory, Leiden University, P.O. Box 9513, RA Leiden 2300, The Netherlands; # Laboratory for Synchrotron Radiation and Femtochemistry, Paul Scherrer Institute, Villigen 5232, Switzerland; ¶ Institute for Modeling Plasma, Atmospheres, and Cosmic Dust (IMPACT), University of Colorado, Boulder, Colorado 80303, United States

## Abstract

The threshold photoelectron spectrum, dissociative photoionization, and pyrolysis of aziridine, a nitrogen-bearing hydrocarbon of c-C_2_H_4_NH composition, were studied by double-imaging photoelectron photoion coincidence spectroscopy at the Swiss Light Source. The aziridine adiabatic ionization energy (AIE) was measured at (9.30 ± 0.03) eV. The full ground-state band of the threshold photoelectron spectrum, exhibiting strong anharmonicity, was simulated using the Thawed Gaussian Approximation (TGA) method. At higher photon energies, dissociative photoionization products corresponding to hydrogen loss and methyl loss were identified with respective appearance energies of (10.59 ± 0.05) eV and (10.53 ± 0.05) eV obtained by fitting a statistical model to the dissociative ionization data. Aziridine pyrolysis was also studied at a temperature of 1360 K. Isomerization plays an important role with multiple C_2_H_5_N isomers contributing to the thermal decomposition of neutral aziridine. Ethenamine, methyl radical, and radicals of H_2_CN^•^ composition were detected as pyrolysis products. The AIE for ethenamine was measured at (8.16 ± 0.03) eV. Isomerization to (*E*/*Z*)-ethanimine and *N*-methylmethanimine was also implicated in the pyrolysis data.

## Graphical abstract

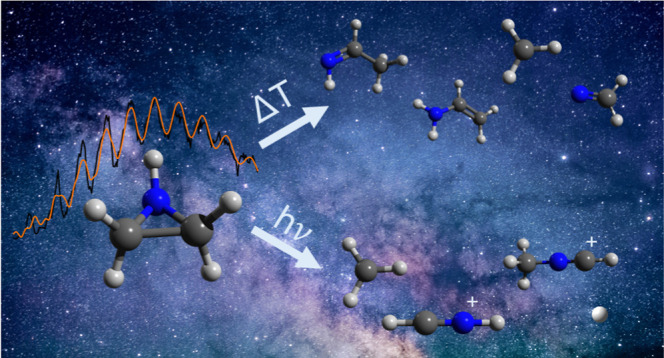

## Introduction

Small heteroatom-containing hydrocarbons are intermediates on the path toward chemical complexity in the interstellar medium (ISM). (*E*/*Z*)-Ethanimine (**E/Z-EAN**) are two such nitrogen-substituted hydrocarbons identified in the Sagittarius B2 North molecular cloud.^1^ Heterocycles are a class of heteroatom-containing hydrocarbons that also form under interstellar conditions. For example, oxirane (c-C_2_H_4_O), the smallest oxygen-containing heterocycle, has been detected in the ISM at higher than expected abundances.^2^ Aziridine (**AZR** c-C_2_H_5_N) is an isomer of **E/Z-EAN**, and is the nitrogen-containing analog of oxirane. Although **AZR** has not yet been unambiguously identified in the ISM, several transitions in radio observations have tentatively been assigned to it.^3–5^

**AZR** and its isomers (see Figure 1) could play a role in the planetary satellite chemistry in our solar system, as well. For example, low-temperature chemistry driven by small heteroatom-substituted hydrocarbons leads to the formation of complex N-bearing hydrocarbons in the atmosphere of Titan,^6,7^ which will be investigated by the Dragonfly mission, intended to launch in 2028. The search for small heteroatom-substituted hydrocarbons is relevant in the context of water ice covered moons as well. Europa Clipper was launched in 2024 and will study the habitability of Jupiter’s ice-covered moon Europa. Nitrogen, an indicator of habitability, has yet to be unambiguously detected on Europa.^8^ Clipper and Dragonfly both feature a suite of scientific instruments to study these two moons. The surface dust analyzer (SUDA)^9^ and mass spectrometer for planetary exploration (MASPEX)^10^ onboard Clipper are two such instruments that require a detailed understanding of the mass spectrometry of nitrogen-containing hydrocarbons to interpret their results fully. To understand how the **AZR** system may interact with these various environments, detection techniques, and potential role in the formation of more complex hydrocarbons, it is important to characterize both the thermal reactivity of **AZR** and the unimolecular reactivity of its cation.

**Figure 1:**
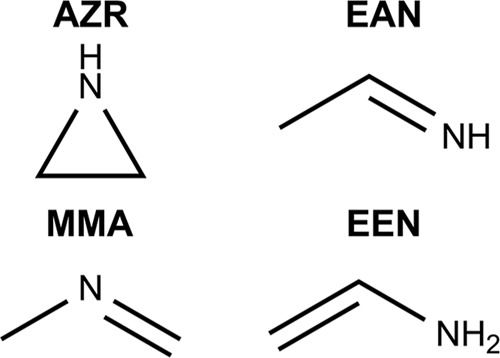
Structures of the C_2_H_5_N isomers aziridine (**AZR**), ethanimine (**EAN**), *N*-methylmethanimine (**MMA**), and ethenamine (**EEN**).

To investigate the thermal stability of **AZR** and related compounds, flash pyrolysis is used here to induce high temperatures on a short time scale. Various groups have studied the pyrolysis of the C_2_H_5_N isomers **AZR**, **EAN**, and **EEN**. However, these studies have only detected closed-shell products, such as hydrogen cyanide, acetonitrile, methane, and C_2_H_3_N isomers, and did not apply spectroscopic approaches to enable isomer-specific identifications.^11–15^ In comparison, the short time-scale of flash pyrolysis and collision-free molecular beam sampling allow for the detection of primary (radical) decomposition products. Because these primary products may go on to react with the most abundant reaction partners, these insights are relevant in high-pressure environments too. For example, pyrolysis of the C_2_H_5_N system has also been investigated in the context of combustion. Specifically, the diradical species H_2_C^•^–NH–CH_2_^•^ (**INT1**) was proposed as an intermediate in pyrrolidine combustion, with **AZR**, **MMA**, and **EEN** detected as stable products.^16–18^ Yet, the experimental results from these studies focused primarily on isomerization pathways of 43 amu species, with less emphasis on subsequent dissociation steps.

Photoion mass-selected threshold photoelectron spectroscopy (ms-TPES) is used here to analyze the complex mixture of pyrolysis products. By probing the ms-TPES of each intermediate and product peak, it is often possible to determine the isomeric composition of pyrolysis products based on reference spectra or Franck–Condon simulations.^19^ The photoelectron spectra of small molecular systems with N–H functional groups with an inversion mode are often poorly reproduced within the harmonic approximation, such as that of ammonia^20^ and methanimine.^21^ It is therefore necessary to benchmark the simulation method against a well-resolved **AZR** photoelectron spectrum first. Previous photoelectron spectroscopic studies of **AZR** have not resolved vibronic structure in sufficient detail.^22,23^ The newly recorded vibrational structure of the **AZR** photoelectron spectrum will allow for a comparative study of its computational modeling as well as a thorough analysis of *m*/*z* 43 ms-TPES in the pyrolysate to provide insight into **AZR** thermal isomerization.

For investigating the unimolecular reactivity of the **AZR** cation as a function of internal energy, we turn to dissociative photoionization (DPI). DPI is governed by the cationic potential energy surface (PES) and the kinetically allowed fragmentation channels as a function of energy.^24^ Previous attempts to understand the cationic behavior of C_2_NH_5_^+^ either neglected reaction barriers,^25^ or focused on a limited portion of the PES.^26^ By studying the experimental appearance energies of fragmentation products, quantum chemical calculations of the cationic PES can be validated. The cations formed in this study are also important to identify due to the enhanced reaction rates of ions in the ISM compared to neutral–neutral reactions.

In this work, we investigate the photoionization, dissociative photoionization (DPI), and pyrolysis of **AZR**. First, the experimental **AZR** mass-selected threshold photoelectron spectrum (ms-TPES) is presented and simulated using various computational chemistry approaches. Second, threshold DPI mass spectra and the breakdown diagram are presented and simulated together with the calculated cationic C_2_H_5_N PES. Last, the pyrolysis products are investigated and rationalized based on their ms-TPES and the neutral C_2_H_5_N PES.

## Methods

### Experimental Section

The (dissociative) photoionization and pyrolysis of **AZR** were studied using the *i* ^2^PEPICO endstation at the Vacuum Ultraviolet (VUV) Beamline of the Swiss Light Source (SLS). The beamline and the experimental setup are briefly described here. Further details on the endstation, beamline, and the Chen-type pyrolysis microreactor can be found in the literature.^27–31^ **AZR** (*m*/*z* 43) was synthesized as described in the Supporting Information of Hicks et al.^32^

For the (dissociative) photoionization studies, **AZR** vapor was seeded into the detection chamber of the *i* ^2^PEPICO spectrometer at room temperature using a needle valve connected to a Teflon tube, resulting in an effusive flow. The flow was set to maintain a static pressure of 3 × 10^–7^ mbar. **AZR** vapor was intersected by the tunable VUV radiation of the beamline. Synchrotron radiation was generated using a bending magnet and dispersed on a 150 grooves per millimeter grating. Depending on the photon energy, either a MgF_2_ window (from 8.0 to 8.6 eV) or a Kr/Ar/Ne gas filter (from 9.1 to 10.9 eV) was used to remove higher grating orders of light. Upon valence ionizing a molecular species, a photoelectron and a photoion are generated. They were accelerated in opposite directions by an electrostatic field of 217 V/cm. Both ions and electrons were velocity map imaged using RoentDek delay line detectors, with the detection of an electron serving as time zero for the time-of-flight measurement of the coincident ion. An all-electron mass spectrum can then be constructed by considering all ions in delayed coincidence with all electrons, which indicates all detected molecular species that ionize at or below the selected photon energy.

Threshold electrons, generated with no prompt kinetic energy, are focused onto the center of the detector. A small ring area around the center can be used to correct for contributions by hot electrons in the center, i.e., electrons with nonzero kinetic energy but with a negligible off-axis momentum that are, therefore, also detected there.^33^ Threshold ionization mass spectra are obtained by subtracting the ring ion counts from the center ion counts in an area-normalized way. Coincidence count rates between threshold electrons and ions of a certain *m*/*z* are then plotted as a function of photon energy to obtain the ms-TPES. Data for the *m*/*z* 43 ms-TPES (**AZR**) were recorded from 9.1 to 10.1 eV with a step size of 5 meV and signal averaging for 100 s per data point. DPI data were signal averaged for 100 s per point from 10.1 to 11.5 eV with a step size of 10 meV. Points with a longer integration time of 1000 s were also recorded from 10.5 to 10.9 eV in steps of 0.1 eV to reveal metastable fragment ion peak shapes and, thus, the dissociation kinetics.

A breakdown diagram is constructed to analyze **AZR** DPI by plotting fractional parent and daughter ion abundances in threshold ionization for photon energies between 10.1 and 10.9 eV. Asymmetric fragment ion peak shapes indicate slow dissociation in the acceleration region of the mass spectrometer. In other words, the metastable parent ion has enough energy to dissociate yet does not do so instantaneously, resulting in a slightly longer than nominal time-of-flight for the fragment ion. If dissociation is slow at threshold compared with the detection time scale, or if it is substantially slower than another fragmentation step, its product ion will not be detected at its threshold energy, but only after some excess energy is also available to the system. This shift of the fragment ion breakdown curve to higher energies is called the kinetic and the competitive shift, respectively. Metastable fragment ion TOF shapes were modeled to extract a rate curve, which is then extrapolated to the 0 K appearance energy within a statistical framework. Parent ion counts at higher photon energies (≥10.9 eV) are highly sensitive to the area-normalizing subtraction factor due to the large number of hot electrons with few threshold ion counts given the low Franck–Condon factors at these energies. For example, one minor artifact of this can be seen in the reemergence of the parent ion signal at higher energies.

For the pyrolysis study, **AZR** was entrained in a flow of argon expanding through a 200 μm pinhole into an approximately 3 cm long Chen-type SiC microreactor, the second half of which was heated resistively using a DC voltage. The **AZR** sample was contained in a glass bubbler outside of the vacuum system. The vapor was picked up using a flow of argon then put through a mass flow controller, resulting in a flow of 20 sccm containing approximately 10% **AZR**  based on its vapor pressure.^34^ A pyrolysis power of 55 W was applied to the SiC microreactor corresponding to a temperature of approximately (1360 ± 100) K based on previous thermocouple measurements on the reactor. The gas flow containing any remaining **AZR** and the pyrolysis products expanded into the source vacuum chamber maintained at a pressure of 10^–4^ mbar by two turbomolecular pumps. A 2 mm diameter skimmer sampled the gas expanding from the reactor as it entered the i^2^PEPICO detection chamber, where VUV light from the beamline intersected the molecular beam. Pyrolysis data were collected from 8.0 to 8.6 eV and from 9.1 to 11.0 eV with a step size of 10 meV and averaging for 140 s per point. Threshold ionization mass spectra are collected at each step in the same manner as described above. Ms-TPES of the **AZR** pyrolysis products were plotted as the threshold ionization ion intensity of a particular *m*/*z* peak as a function of VUV photon energy. Despite the rich mixture, we saw no evidence of bimolecular chemistry taking place in the reactor (see below and in Supporting Information).

### Computational

Quantum chemical calculations were performed using the Gaussian 16 suite of programs^35^ on the Alpine cluster at the University of Colorado Boulder. Density functional theory (DFT) was used for exploring the PES of neutral and cationic **AZR** with the M06-2X functional and aug-cc-pVTZ basis set.^36^ Transition states were confirmed using intrinsic reaction coordinate (IRC) scans to ensure they connect the intended minima. Dissociation pathways were not found to have reverse barriers at the DFT level of theory except where shown. Stationary point energetics were then refined at the full CBS-QB3 level of theory.^37^ Energies at the CBS-QB3 level were also used to verify adiabatic ionization energies (AIEs) after comparison with CCSD­(T) calculations extrapolated to the complete basis set limit as discussed in the Supporting Information. Due to lower computational expense, CBS-QB3 was chosen for further calculations in this work. Harmonic calculations including CCSD­(T)/aug-cc-pVTZ, CCSD/aug-cc-pVTZ, and M06-2X/aug-cc-pVTZ were used as the basis of Franck–Condon stick spectra computed within the double-harmonic (adiabatic Hessian) approximation using Gaussian 16. As the origin transition connects the mostly harmonic vibrational ground states of the neutral and the cation, experimental AIEs were assigned by matching the double harmonic approximation origin bands to the corresponding peaks in the experimental ms-TPES, whenever these were observed. Further details and the corresponding double-harmonic approximation spectra can be found in the Supporting Information.

Comparison between the simulated double-harmonic spectra and the experimental data shows that the harmonic approximation is not sufficient to reproduce the vibronic structure of the photoelectron spectrum of aziridine correctly. This is mainly due to the double-well shape of the cationic potential energy surface along the N–H bending coordinate, giving rise to strong anharmonic effects. Therefore, the spectrum was calculated using the Thawed Gaussian Approximation (TGA), originally proposed by Heller^38^ and successfully employed for the simulation of vibrationally resolved emission^39^ as well as photoelectron spectra^20,40^ of molecules with double-well potential energy surfaces. We used a modified version of an in-house software package originally developed for the simulation of photoemission spectra and the calculation of internal conversion rates.^41^ In brief, the TGA method is based on solving the time-dependent Schrödinger equation for a wavepacket always confined to a Gaussian shape. This way, the computational effort is reduced to solving a coupled set of equations for the wavepacket center, momentum, width, and phase. The temporal change of center and momentum follow classical-like equations, which allows the simulation to be based on the propagation of a molecular dynamics trajectory with the nuclear forces computed “on the fly.” The trajectory follows a path on the full-dimensional anharmonic potential energy surface. We propagated the trajectory over 200 fs with a time step of 0.2 fs. To obtain the time-dependent width and phase of the wavepacket, the Hessian matrix of the second derivatives of the electronic potential energy with respect to the nuclear coordinates is also required along the trajectory and is calculated every 1.0 fs. In our case, the energies, gradients, and Hessians have been computed at the ωB97X-D/aug-cc-pVTZ level of theory using Gaussian 16 as this functional was found to better reproduce the experimental data than M06-2X (see Supporting Information). While the neutral and cation geometry optimization and harmonic frequency analysis for the double harmonic Franck–Condon calculation is feasible using coupled cluster singles and doubles, the large number of energy, gradient, and Hessian calculations along the trajectories requires a switch to a DFT level of theory for the TGA simulations.

For simulating photoelectron spectra, the TGA trajectory is initiated in the cationic ground state at the neutral optimized geometry with the initial momenta set to zero and the wavepacket width chosen to match the vibrational ground state of the neutral species. The initial and the time-evolved Gaussian wavepacket are used to compute the autocorrelation function, which is subsequently Fourier transformed to yield the photoelectron spectrum. The energetic position of the spectrum is obtained by including the vertical ionization energy (VIE) in the autocorrelation function.

As DFT methods are known to provide ionization energies of varying accuracy due to their treatment of exchange and correlation, it is necessary to shift our TGA spectra. To ensure invariance to the choice of ionization energy, the simulated spectra shown correspond to only the Fourier transform of the autocorrelation function rather than the photoionization cross section. As the TGA method best reproduces spectral structure near its maximum values, the TGA spectra of **AZR** and **EEN** were offset by 0.139 and 0.218 eV, respectively, to provide the best match with the VIE observed in the experimental ms-TPES. For **MMA**, **E-EAN**, and **Z-EAN**, where the origin transition has negligible cross section and there is ambiguity in the isomeric assignment, the simulated spectra were instead anchored to the CBS-QB3 calculated VIEs. VIEs, AIEs, and the relative shifts from ωB97X-D/aug-cc-pVTZ to CBS-QB3 VIEs for all TGA simulated spectra can be found in the Supporting Information.

Modeling of the **AZR** DPI was accomplished using the miniPEPICO program, which has been described in detail in the literature.^42^ Briefly, computed vibrational frequencies and rotational constants for neutral **AZR** were used to calculate the thermal energy distribution, which is shifted by the ionizing photon energy minus the adiabatic ionization energy into the ion manifold. Microcanonical RRKM fragmentation rate constants as a function of internal energy depend on the density of states of the dissociating species and the number of states of the transition state. The density of states was calculated for the deepest well active in the dissociation, that of the cationic intermediate **INT1** ^**+**^. The number of states of the transition states were computed based on harmonic ab initio vibrational frequencies. Ion optics parameters and the experimental temperature are then used to simulate the breakdown curve and the TOF distributions. In the fit, the experimental data are best reproduced by varying the DPI thresholds (0 K appearance energies) and by scaling the lowest five vibrational modes of the transition states to adjust the activation entropy and fit the model to the measured rate curve.

## Results

### Threshold Photoelectron Spectroscopy

The experimental ms-TPES of **AZR** is shown in black in Figure 2. The first strong transition is observed with a maximum at (9.30 ± 0.03) eV, which is assigned as the origin transition due to the favorable comparison with the double harmonic CCSD/aug-cc-pVTZ simulated spectrum (see Figure S1a). The experimentally assigned AIE is supported by the CBS-QB3 calculated AIE of 9.33 eV and the literature value of 9.28 eV.^43^ The double harmonic approximation describes the first few transitions in the experimental spectrum near the origin transition, where the potential is least anharmonic, relatively well. However, results at different levels of theory diverge and fail to reproduce the vibronic band structure at higher energies (see Supporting Information). This is attributed to the double-well along the N–H inversion mode on the cationic **AZR** PES. To account for anharmonic effects in the **AZR** spectrum, the TGA method was used with the resulting simulated photoionization spectra shown in orange in Figure 2. The time-dependent TGA method reproduces the ground-state band in the experimental ms-TPES very well because of its ability to address the double-well potential of **AZR**. The TGA method captures the vibronic structure near the vertical ionization energy (VIE) best, resulting in excellent agreement near the maximum of the vibronic structure. By combining the vibronic structure from the TGA method and the AIE assigned through the double harmonic approximation, we can explain the observed spectrum.

**Figure 2:**
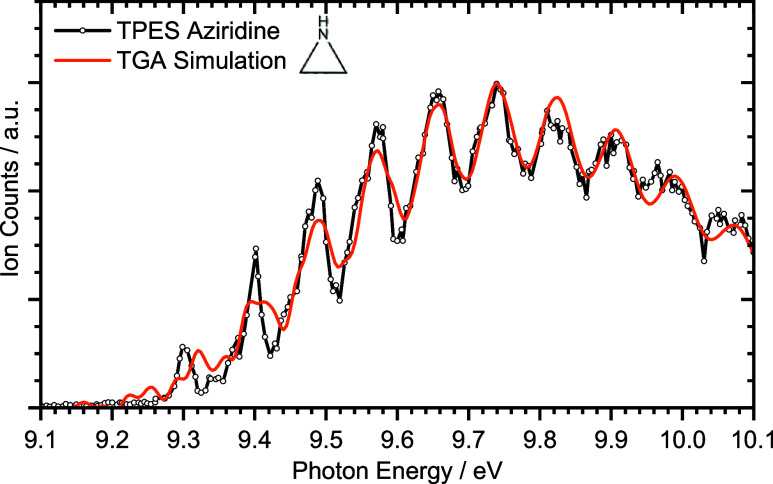
Ms-TPES of room-temperature **AZR** (black, *m*/*z* 43) in the photon energy range of 9.1–10.1 eV compared to the TGA-simulated spectrum at the ωB97X-D/aug-cc-pVTZ level of theory (orange) which has been offset as described in the Computational Methods. A krypton resonance line from the gas filter is seen at 10.04 eV.

With TGA, the vibronic structure of the spectrum corresponds to recurrences of the wavepacket autocorrelation function in the time domain. The oscillation frequency associated with the recurrence time corresponds to an energy difference of 0.086 eV, which fits the experimentally observed peak spacing of the spectrum (see Figure 2). Insights regarding the active vibronic normal modes can be gleaned from inspecting the change of the molecular structure along the trajectory when significant wavepacket recurrences occur. Figure 3 shows the change in molecular structure of **AZR** together with the electronic energy and the absolute value of the autocorrelation function. The main recurrence responsible for the spectral shape occurs after around 48 fs and is due to an oscillatory wagging motion of the N–H bond. This motion is confined to one of the minima of the double-well potential. Although the trajectory samples the potential energy surface at energies as high as 0.5 eV (see Figure 3a) and the inversion barrier in the cation is only 0.088 eV, the wavepacket stays localized in a single well within 150 fs. Wavepacket confinement is due to the trajectory not passing close enough to the minimum energy inversion path. On longer time scales, the wavepacket does reach the other minimum, which leads to a vanishing autocorrelation function and thus does not influence the modeled spectrum (see Supporting Information). For a more detailed discussion of the vibrational structure, please see the Supporting Information.

**Figure 3:**
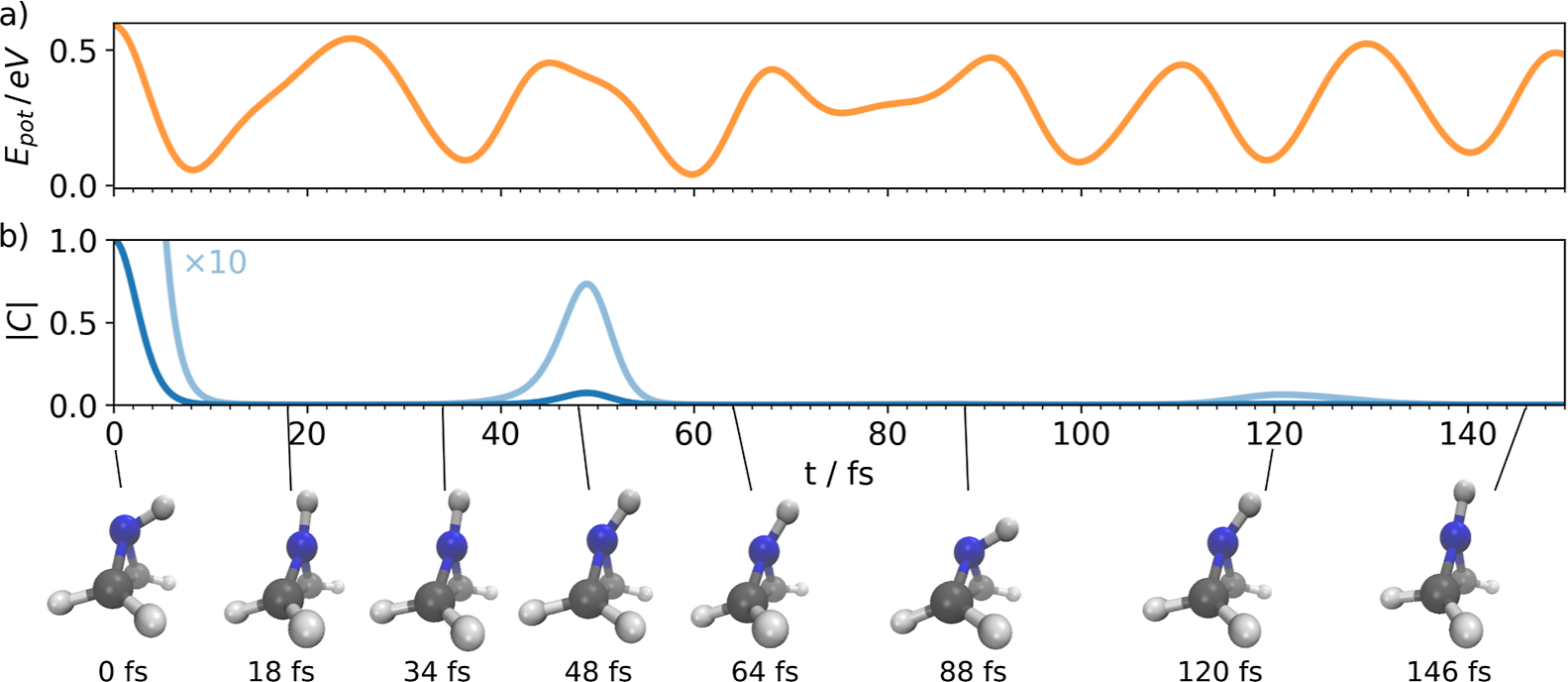
(a) Electronic potential energy along the TGA trajectory of aziridine cation obtained at the ωB97X-D/aug-cc-pVTZ level of theory. The zero energy level corresponds to the optimized cation energy. (b) Absolute value of the wavepacket autocorrelation function along the trajectory. For representative instants of time, the molecular structure is shown, illustrating the N–H wagging motion.

### Dissociative Photoionization

A series of threshold photoionization mass spectra from 10.5 to 10.9 eV photon energy is shown in Figure 4. Daughter ions are observed at *m*/*z* 42 and 28. The time-of-flight signal observed at *m*/*z* 28 is asymmetric at lower photon energies, indicative of slow dissociation of the parent ion after ionization in the acceleration field of the mass spectrometer. The *m*/*z* 28 signal can be attributed to either an isomer of CH_2_N^+^ composition or the ethylene cation (C_2_H_4_^•+^). The signal at *m*/*z* 42 is of C_2_H_4_N^+^ composition as the result of hydrogen atom loss from the parent ion. As seen in Figure 4, both DPI daughter ion signals become more symmetric as the internal energy increases indicating that the parent ion has sufficient internal energy to dissociate rapidly in the ionization region. The breakdown diagram of **AZR** is shown in Figure 5.

**Figure 4:**
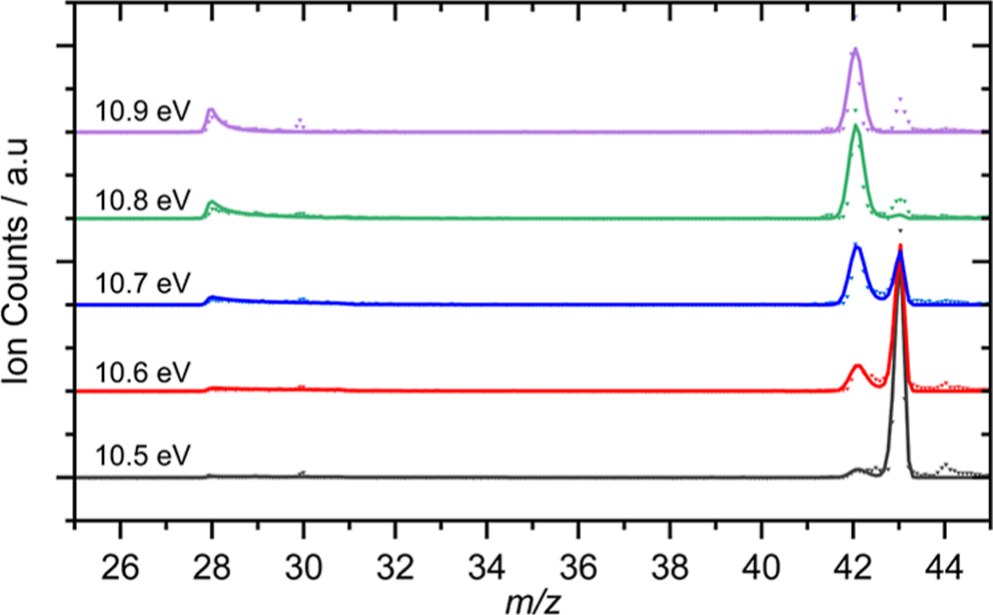
Threshold ionization mass spectra of room temperature **AZR** at representative photon energies using 1000 s integration time (triangles). Continuous lines represent the statistical fit to the experiment. A minor formaldehyde contaminant signal can be seen at *m*/*z* 30.

**Figure 5:**
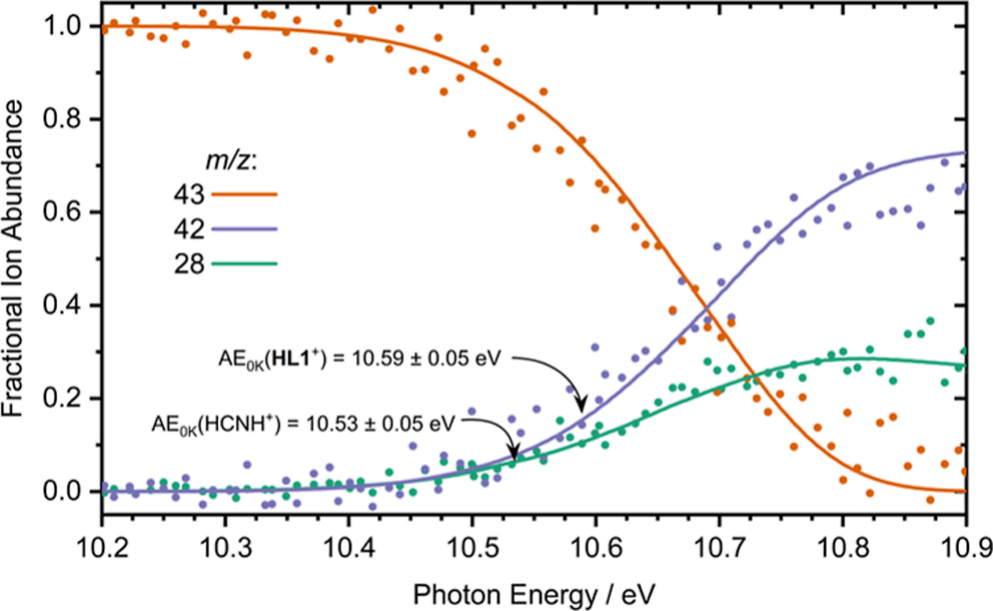
**AZR** breakdown diagram (dots). Also shown are the simulated breakdown curves (lines) generated using a statistical model.

Quantum chemical calculations on the C_2_H_5_N^•^^+^ PES were guided by the experimental observations to reveal the **AZR** DPI mechanism. Stationary points along the PES calculated using CBS-QB3 are depicted in Figure 6. Only the lowest energy pathway for each dissociation route is depicted here (see Supporting Information for more). The lowest energy pathway starting from the **AZR** ^+^ radical cation follows the opening of the C–C bond over **TS1** ^**+**^ at 9.75 eV to form **INT1** ^**+**^. **INT1** ^**+**^ can undergo a hydrogen transfer either from the NH to the CH_2_ group to form **MMA** ^**+**^ at 8.58 eV over **TS2** ^**+**^ at 10.59 eV, or from one CH_2_ group to the other over **TS3** ^**+**^ at 10.53 eV to form (methylamino)­methylene (**MAM** ^**+**^) at 8.88 eV. The lowest energy dissociation mechanism of **MMA** ^**+**^ involves a hydrogen loss over **TS4** ^**+**^ at 10.06 eV to form **HL1** ^**+**^. The lowest energy dissociation of **MAM** ^**+**^ goes over **TS5** ^**+**^ at 10.09 eV, resulting in the formation of methyl radical and HCNH^+^ (*m*/*z* 28) at 9.90 eV. Direct hydrogen loss from **AZR** to **HL2** ^**+**^ requires 11.22 eV and is beyond the energy range of our work.

**Figure 6:**
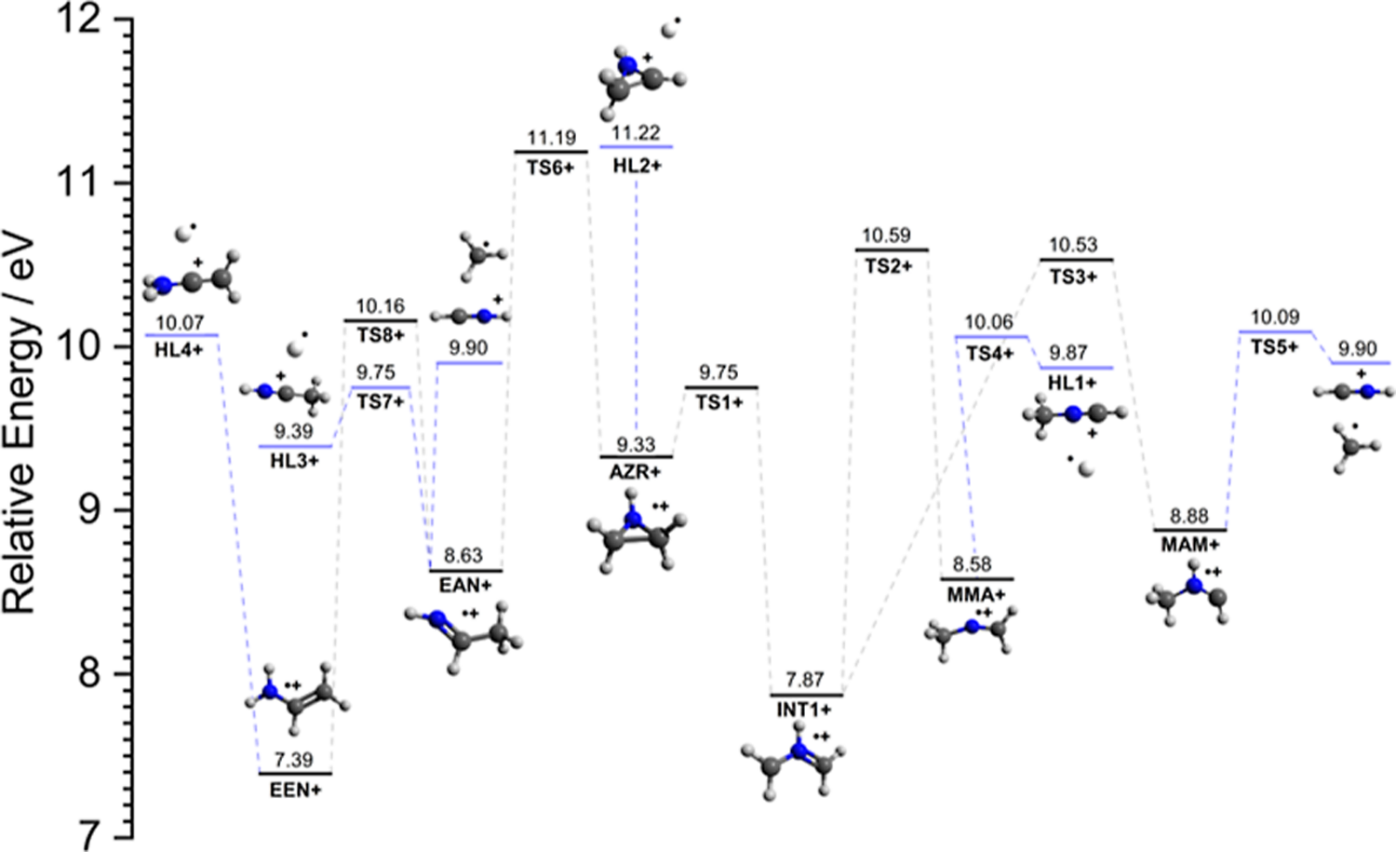
C_2_H_5_N^•^^+^ PES at the CBS-QB3 level of theory. All energies are reported in eV relative to **AZR**. Black lines indicate isomerization pathways while blue lines indicate dissociation pathways.

Another isomerization pathway from **AZR** ^**+**^ involves transferring a hydrogen from one CH_2_ group to the other, followed by C–N bond breaking over **TS6** ^**+**^ at 11.19 eV to form **E-EAN** ^**+**^. The pathway to **E-EAN** ^**+**^ is shown, which can freely interconvert to the isoenergetic **Z-EAN** ^**+**^ at 8.63 eV over a barrier of only 1 meV. **E-EAN** ^**+**^ can then lose a hydrogen over **TS7** ^**+**^ at 9.75 eV to form **HL3** ^**+**^, or it can lose methyl radical to form HCNH^+^ at 9.90 eV. **TS6** ^**+**^ also acts as the rate-limiting transition state to form **EEN** ^**+**^ by hydrogen migration from the methyl group of **EAN** to the amine over **TS8** ^**+**^ at 10.16 eV. **EEN** ^**+**^ can then lose a hydrogen at 10.07 eV to form **HL4** ^**+**^. Overall, **TS6** ^**+**^ as a rate-limiting transition state lies significantly above the energy range used for our DPI studies, however, this portion of the potential energy surface was explored because of its relevance in pyrolysis (see below). Based on the PES, DPI products of *m*/*z* 42 and 28 result from **MMA** ^**+**^ hydrogen loss and **MAM** ^**+**^ methyl loss with calculated rate-limiting barriers of 10.59 and 10.53 eV respectively.

The calculated energies and the harmonic vibrational normal modes corresponding to the rate-limiting transition states, **TS2** ^**+**^ and **TS3** ^**+**^, are used as initial guesses in the statistical model to reproduce the experimental breakdown diagram and selected TOF distributions. The resulting model is shown as the solid lines in Figure 5 and predicts 0 K appearance energies of (10.59 ± 0.05) eV for hydrogen loss (*m*/*z* 42) and (10.53 ± 0.05) eV for methyl loss (*m*/*z* 28). The excellent agreement between calculated and experimental values supports the proposed mechanism.

### Pyrolysis

The ms-TPES of *m*/*z* 43 changes fundamentally upon pyrolysis as seen in Figure 7. Whereas **AZR** has an AIE of 9.30 eV, the *m*/*z* 43 ms-TPES following pyrolysis rises already at ca. 1 eV lower photon energies. The magnitude of this shift, together with the observed vibrational structure, rules out hot-band contributions and supports **AZR** isomerization in pyrolysis. The TGA spectrum of **EEN** reproduces the postpyrolysis *m*/*z* 43 ms-TPES onset remarkably well. Together with the harmonic Franck–Condon simulation (Figure S9), we assign the AIE of **EEN** as (8.16 ± 0.03) eV, in agreement with the CBS-QB3 calculated AIE of 8.14 eV. This confirms **EEN** as a thermal isomerization product of **AZR**. The TGA simulated spectrum of **EEN** is dominated by the amine wagging mode, which, in contrast to **AZR**, has no double-well character. An analysis of the nuclear dynamics revealed by the TGA simulation is presented in the Supporting Information

**Figure 7:**
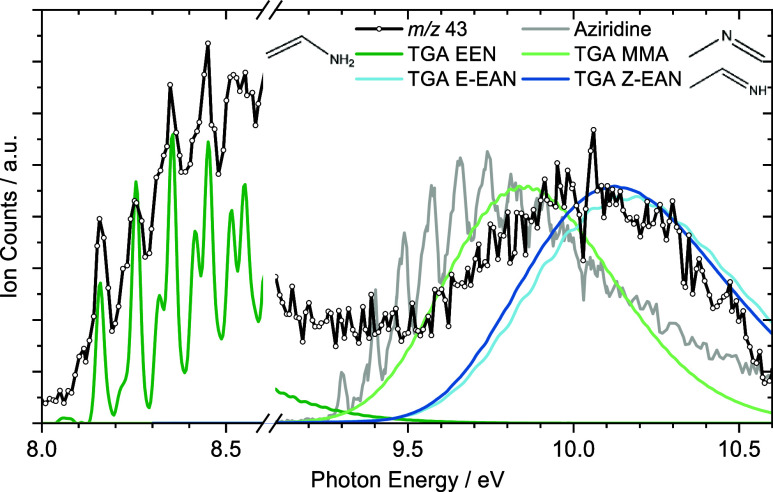
Ms-TPES of *m*/*z* 43 from 8.0 to 8.6 eV and from 9.1 to 10.6 eV in the pyrolysis of **AZR** at 1360 K (black), compared to the room-temperature **AZR** ms-TPES (gray). TGA simulations of **EEN**, **MMA**, **Z-EAN**, and **E-EAN** at the ωB97X-D/aug-cc-pVTZ level of theory are shown and are shifted as described in the Computational Methods. A krypton resonance line from the gas filter is seen at 10.04 eV.

Additionally, the **AZR** vibronic bands are no longer visible in the 9 to 10 eV photon energy range. Instead, a broad feature is observed with a maximum at 10.1 eV. Three isomers could be the origin of the broad feature observed: **Z-EAN**, **E-EAN**, and **MMA**. These three species all have ionization energies within the studied range, calculated as 9.52 eV for **E-EAN**, 9.50 eV for **Z-EAN**, and as 9.11 eV for **MMA** using CBS-QB3. These values are also in agreement with the reported literature onsets of 9.5 eV for **EAN** isomers and 9.3 eV for **MMA**.^44^ At least one of these isomers likely contributes as the first cationic excited state of **EEN** does not appear until 11 eV.^45^ TGA simulated spectra of these isomers all yield broad, unstructured vibronic bands of approximately 1 eV width, in line with experiment (Figure 7). In the absence of vibrational fine structure, a conclusive isomer-specific assignment of this signal is not possible based on the ms-TPES results alone.

The pyrolysate mass spectra at a photon energy of 10 eV shown in Figure 8 exhibit new peaks at *m*/*z* 15, 28, and 42. Besides trace contaminants, no higher mass species are seen that would indicate bimolecular chemistry. A plot demonstrating integrated ion intensity as a function of temperature for each observed product can be found in the Supporting Information. The isomeric nature of the pyrolysis products was also investigated through the ms-TPES for each of the detected species as shown in Figure 9. The ms-TPES of *m*/*z* 15 at a pyrolysis temperature of 1360 K exhibits a strong resonance at 9.8 eV matching the literature TPES of the methyl radical and confirming the presence of neutral methyl in the pyrolysate.^46^

**Figure 8:**
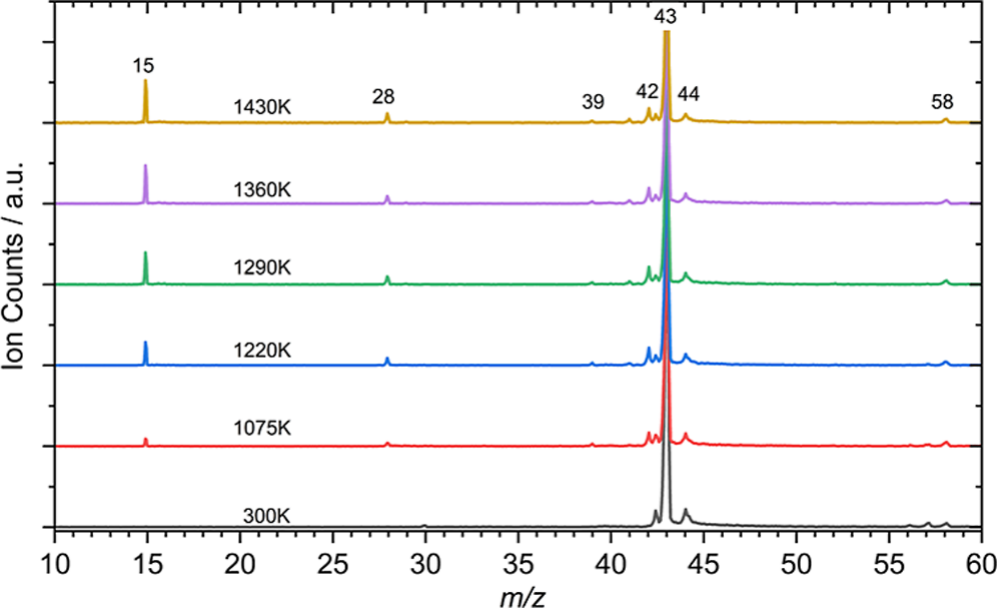
**AZR** pyrolysis mass spectra at 10.0 eV photon energy as a function of reactor temperature. Minor propargyl and acetone contaminants are seen at *m*/*z* 39 and *m*/*z* 58, respectively, from a previous experiment.

**Figure 9:**
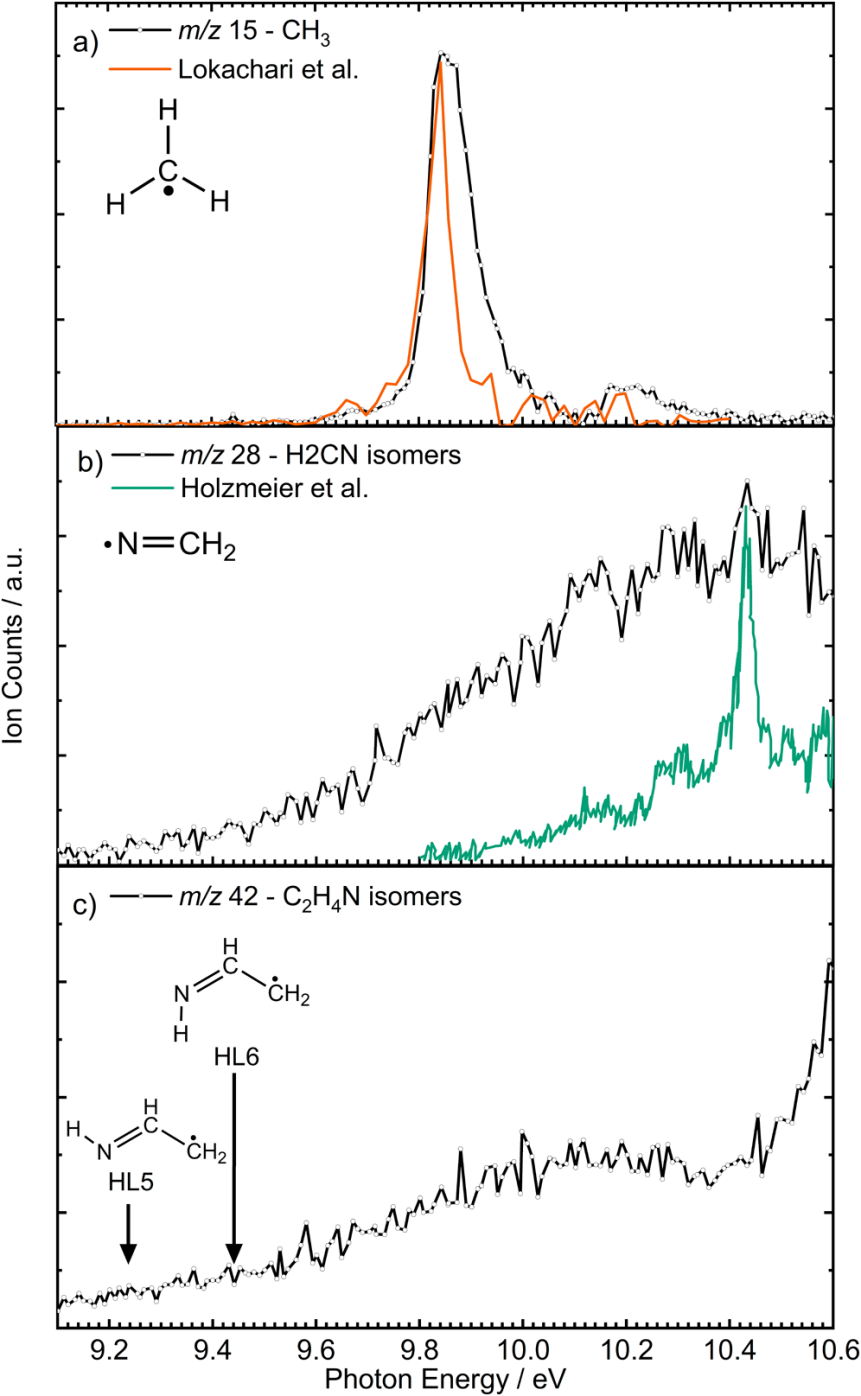
Ms-TPES of **AZR** pyrolysis products recorded at 1360 K across a photon energy range of 9.1 to 10.6 eV with a step size of 10 meV. The ms-TPES correspond to (a) *m*/*z* 15, (b) *m*/*z* 28, and (c) *m*/*z* 42. Reference spectra are reproduced with permission for methyl and a collection of H_2_CN isomers at *m*/*z* 28.^21,46^

The ms-TPES of *m*/*z* 28 does not have a sharp resonance at 10.5 eV, therefore the neutral carrier of this peak is not ethylene.^47^ Instead, *m*/*z* 28 must be of H_2_CN composition. A comparison of our ms-TPES to the literature spectrum for H_2_CN taken by Holzmeier et al.,^21^ which contains H_2_CN, (*E*)-HCNH, (*Z*)-HCNH, and CNH_2_, shows qualitative agreement. There is a difference in the relative intensity of the peak at 10.43 eV. A likely explanation involves the dissociative ionization of **E-EAN**, which may yield HCNH^+^ and a neutral methyl fragment (see Figure 6) at a 0 K appearance energy of ca. 10.8 eV. Because of the large internal energy of the **EAN** formed in pyrolysis, the effective onset of methyl loss in dissociative ionization may red-shift by more than 1 eV, contributing to our pyrolysis ms-TPES of *m*/*z* 28. However, the probability that a neutral DPI fragment, the methyl radical in this case, is ionized by another VUV photon is vanishingly small in the quasi-continuous wave PEPICO experiment. Therefore, the methyl signal at *m*/*z* 15 indicates that at least part of the *m*/*z* 28 signal must be due to pyrolysis production of isomers of H_2_CN composition.

The ms-TPES of *m*/*z* 42 exhibits no fine structure and is likely composed of one or multiple products of hydrogen loss (H_4_C_2_N). Possible sources of this isomer could either be in the hydrogen loss of the *m*/*z* 43 isomers produced by pyrolysis or the DPI of these same isomers. A summary of the calculated AIEs of these isomers can be found in the Supporting Information, with AIEs ranging from 6.2 to 9.44 eV. However, no ion signal is detected at *m*/*z* 42 below 8.6 eV. The identity of this peak is discussed in greater detail below.

To gain insight into the isomerization and dissociation observed in **AZR** pyrolysis, the PES of neutral C_2_H_5_N was explored as shown in Figure 10. The neutral surface exhibits two main initial pathways: formation of **MMA** and **E-EAN**. Of these, the lowest energy pathway identified goes over **TS1** at 1.94 eV to form **INT1**, followed by a rate-limiting hydrogen migration over **TS2** at 2.49 eV to form **MMA**. Most **MMA** will readily dissociate to form methyl radical and H_2_CN^•^ at 2.57 eV, due to the lack of a mechanism for stabilization into the **MMA** well and the similar energetics required for isomerization and dissociation. **INT1** can also form the singlet carbene **1MAM** through a hydrogen migration over **TS3** at 3.09 eV. **1MAM** can lose methyl radical to form E-HCNH^•^ at 2.93 eV.

**Figure 10:**
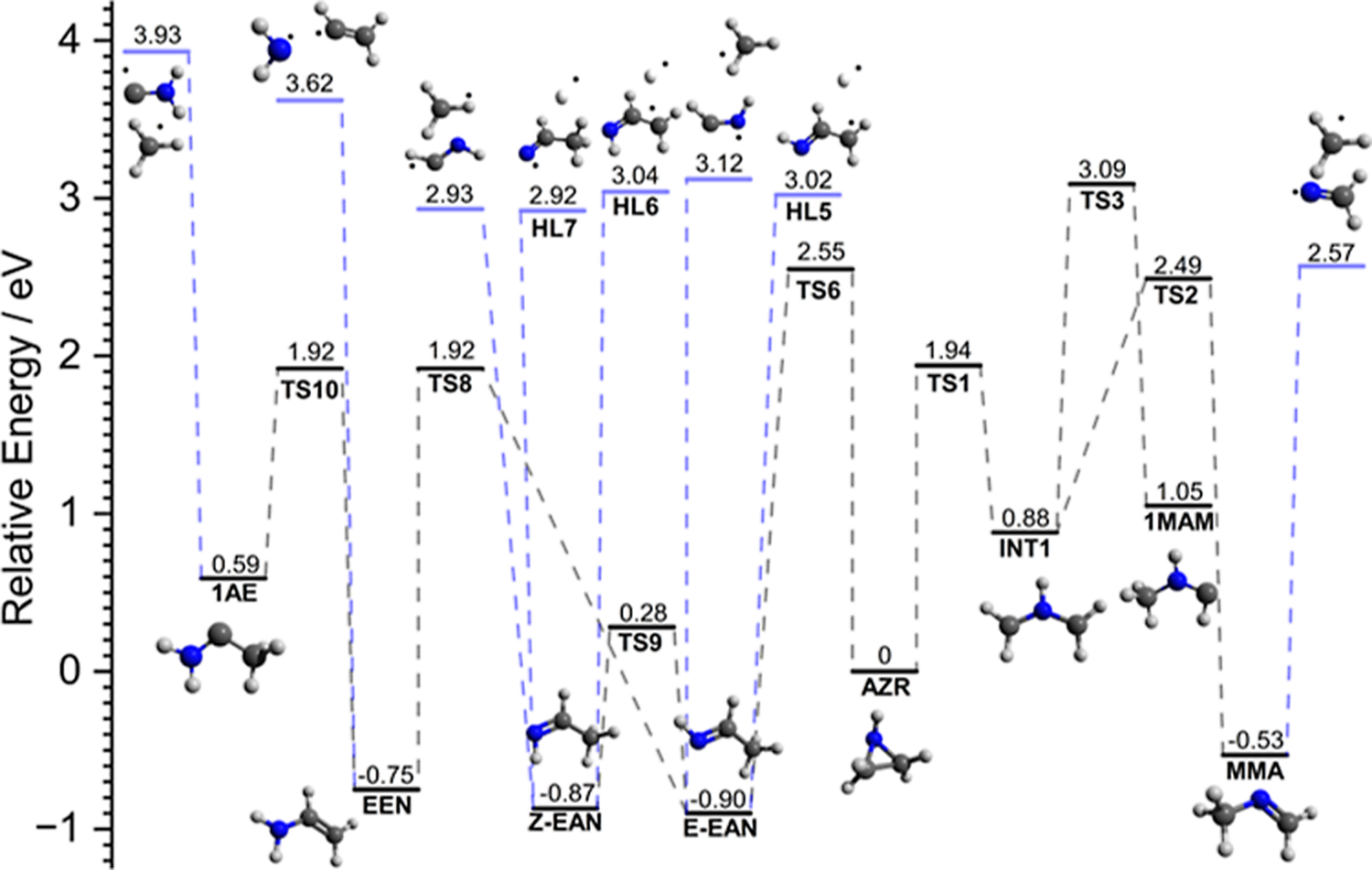
Neutral PES for **AZR** with all energies in eV relative to the ground state of **AZR**. Black lines indicate isomerization pathways while blue lines indicate dissociation.

A competing pathway to **MMA** formation originates from **AZR** with a hydrogen migration from one CH_2_ group to the other, crossing **TS6** at 2.55 eV to form **E-EAN**. From there, **E-EAN** can readily isomerize to **Z-EAN** over **TS9** at 0.28 eV. **E-EAN** can lose methyl radical to form Z-HCNH^•^ at 3.12 eV, while **Z-EAN** can lose the methyl to form E-HCNH·at 2.93 eV. Similarly, **E-EAN** and **Z-EAN** can lose a methyl hydrogen to form **HL5** and **HL6** at 3.02 and 3.04 eV respectively. Alternatively, either isomer of **EAN** can lose the amine hydrogen to form the lowest energy hydrogen loss product, **HL7**, at 2.92 eV. Only the three lowest energy hydrogen loss pathways are discussed here, the energetics of other relevant hydrogen loss isomers can be found in the Supporting Information. **HL5** and **HL6** have calculated AIEs of 9.23 and 9.44 eV, while **HL7** has a calculated AIE of 6.94 eV. No photoion counts were detected at *m*/*z* 42 below 8.6 eV, indicating that **HL7** is likely not present in the pyrolysate. Simulations of the TPES of **HL5** and **HL6** using the TGA method (Figure S11 in Supporting Information) predict sharp vibronic transitions near the origin. The lack of strong peaks in the corresponding portion of the *m*/*z* 42 ms-TPES (Figure 9c) indicates a minor presence of **HL5** and **HL6** in the pyrolysate. From these findings, the signal at *m*/*z* 42 is likely dominated by DPI of hot *m*/*z* 43 isomers with minimal hydrogen loss contributions from neutral 43 amu species in pyrolysis.

The lowest energy pathway to form **EEN** occurs through hydrogen migration from the methyl to the amine group of **E-EAN** over **TS7** at 1.92 eV. **EEN** could dissociate to form vinyl radical and NH_2_ at 3.62 eV, but no signal is observed experimentally at *m*/*z* 27. Further isomerization of **EEN** can yield the singlet carbene 1-aminoethylidene at 0.59 eV (**1AE**) through **TS10** at 1.92 eV. CNH_2_^•^, the last isomer of *m*/*z* 28, could form from dissociation of **1AE** at 3.93 eV, although this pathway is not expected to be competitive given the lack of vinyl detection. On the whole, **EEN** and **EAN** can already be formed in an energy and temperature range where their further fragmentation is still inhibited and the competition between rethermalization and interconversion kinetics will determine their fractional abundance in the pyrolysate.

## Discussion and Conclusion

The ms-TPES of **AZR** was recorded and the AIE assigned at (9.30 ± 0.03) eV. To account for the strong anharmonicities induced by the double-well character of the **AZR** ^+^ PES, the spectrum was simulated using the TGA method. A good match between theory and experiment for the complete ground-state TPES band underscores the ability of TGA to capture such effects. Analysis of the TGA simulation shows that the N–H wagging motion contributes most strongly to the **AZR** vibronic spectra. Rani et al.^23^ have previously reported on the simulation of the photoelectron spectrum of **AZR** including the cationic ground state, cationic excited states, and a full-dimensional quantum dynamics treatment of the vibronic coupling between these states. With this approach, the overall spectral shape of the preexisting valence photoelectron spectrum data could be accounted for. However, possibly due to the use of a restricted subspace of the potential energy surface, the vibrational structure in the ground state band was only partially resolved. Our work complements their findings by presenting the first well-resolved ground-state band in the threshold photoelectron spectrum capable of clearly resolving the anharmonic vibronic structure. We also provide a simulation based on the TGA method that accurately reproduces the vibrational fine structure and explains its physical origin.

Dissociative photoionization of **AZR**, which has not been extensively studied in the literature, was found to yield cationic products at *m*/*z* 42 and 28 below a photon energy of 11 eV. Our PES calculations find that **HL1** ^**+**^ and HCNH^+^ are the ionic products of the lowest-energy pathways to the formation of *m*/*z* 42 and 28 on the cationic surface. Appearance energies of (10.59 ± 0.05) eV and (10.53 ± 0.05) eV were found for *m*/*z* 42 and 28 respectively using an RRKM model to fit both the breakdown diagram and the TOF data. As the formation of **EEN** ^**+**^ and **EAN** ^**+**^ is not competitive across the measured energy range, isomerization of **AZR** ^**+**^ to **MMA** ^**+**^ and **MAM** ^**+**^ drives the formation of these dissociative ionization fragments, with an onset energy significantly above Lyα but much below the ionization energy of hydrogen.

**AZR** flash pyrolysis at 1360 K was found to induce isomerization to **EEN**, confirmed by the measured ms-TPES, TGA simulations, and ionization energy calculations, with an assigned AIE of (8.16 ± 0.03) eV. Other isomers of C_2_H_5_N composition are observed in the ms-TPES of pyrolyzed **AZR** between 9.4 and 10.6 eV. **MMA** is not expected to be a dominant isomerization product in the pyrolysate, because it will readily decompose to H_2_CN^•^ and methyl close to its barrier to formation. **Z-EAN** and **E-EAN** are the most likely isomerization products along with **EEN** given the agreement of their simulated spectra and the observed *m*/*z* 43 ms-TPES, their almost 4 eV stability toward fragmentation, and the expected quasi-equilibrium between **EEN** and **EAN**. Pyrolysis products in the form of H_2_CN^•^ isomers and methyl radical are also detected and confirmed by ms-TPES. NCH_2_^•^ is found to likely be the most abundant H_2_CN^•^ isomer due to the relatively low barrier to its formation, followed by the possible formation of E-HCNH^•^ and Z-HCNH^•^. CNH^•^ and vinyl are too high in energy to be formed. Hydrogen loss isomers, namely **HL5** and **HL6**, cannot be entirely ruled out of the pyrolysate. However, the absence of the most stable hydrogen loss isomer, **HL7**, and the lack of clear vibronic signatures suggests that the *m*/*z* 42 signal can be attributed to DPI following pyrolysis.

Hou et al.^18^ also investigated isomerization on the neutral C_2_H_5_N PES under pyrolysis conditions. Specifically, they assigned the isomers **AZR**, **MMA**, and **EEN** to their photoionization efficiency spectrum for a thermal decomposition product of pyrrolidine at *m*/*z* 43. We see significantly less **AZR** and **MMA** in our pyrolysate as evidenced by the disappearance of the vibronic signature of the former in the ms-TPES and a readily accessible dissociation pathway for the latter. Also, they reported no experimental evidence of further thermal decomposition, as observed in our work. Discrepancies between our findings may result from the different pyrolysis precursors or from different reaction conditions. Nonetheless, the PES/RRKM approach of Hou et al.^18^ supports our finding that hydrogen loss is not a major fragmentation channel. There is also agreement between the PES they present and our neutral PES, with our experimental results ultimately adding more detailed insight into the thermal processing of **AZR**.

From the experimental results in combination with the calculations, it is clear that isomerization plays a crucial role in both the cationic and neutral degradation of **AZR**. Figure 11 shows a summary of the major products produced from DPI and flash pyrolysis of **AZR** in the experimental data. While the neutral surface has similar energetics of ca. 2.5 eV required to access multiple isomerization pathways, the cation favors isomerization to **MMA** ^**+**^ rather than to **EAN** ^**+**^ by 0.6 eV. H_2_CN isomers are identified as both pyrolysis and DPI products. DPI products of **AZR** are formed after isomerization to **MMA** ^**+**^ or **MAM** ^**+**^. In the neutral, isomerization leads to **EEN** and **EAN** isomers, which are stable at 1360 K and the flash pyrolysis time scale, or the system passes through the **MMA** potential energy well to yield CH_2_N^•^ and methyl radical. The differences in product formation observed here help constrain the relevance of each isomer on both the neutral and cationic surface.

**Figure 11:**
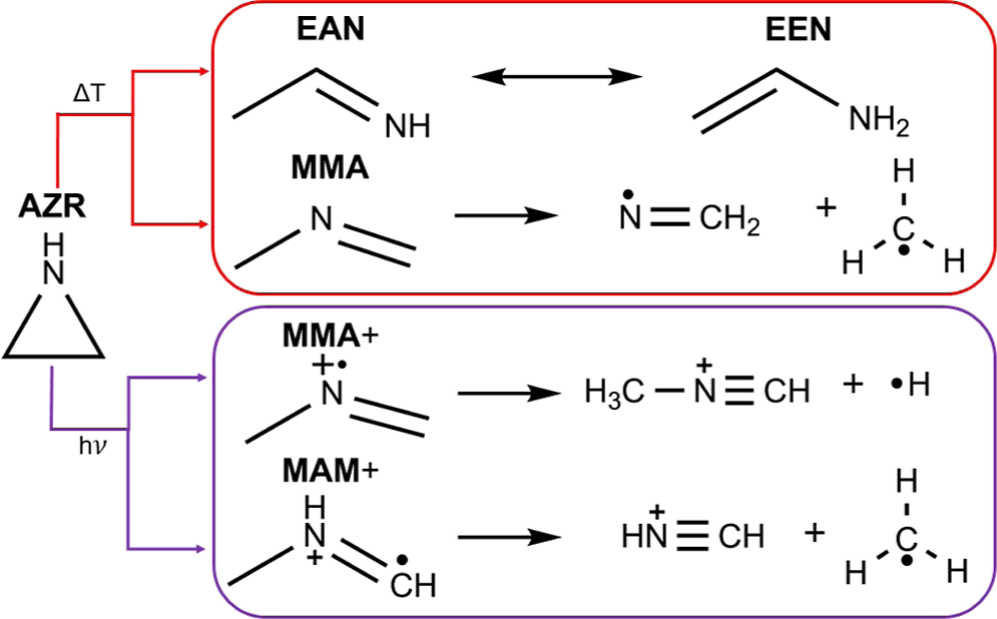
Major reaction pathways for **AZR** following flash pyrolysis at 1360 K (top) and DPI below a photon energy of 11 eV (bottom).

Small, nitrogen-containing hydrocarbons such as the C_2_H_5_N isomers studied here may play an important role in extreme environments, such as the ISM, Titan, and Europa, as reservoirs of heteroatom reactivity. This work helps to unveil the degradation pathways of molecules such as **Z/E-EAN** under pyrolysis and DPI conditions. **AZR** has been shown to be stable with regard to single photon dissociative ionization by Lyα, with higher energies producing fragments of H_2_CN^+^ composition. Interestingly, **AZR** is found to stably isomerize upon pyrolysis to **EEN** and likely **Z/E-EAN** relatively cleanly, possibly enabling further studies into these otherwise reactive species. Lastly, understanding the interplay between these different isomers provides insight into the chemical history of the environments in which they are detected.

## Supplementary Material



## Notes

Calculated structures and vibrational frequencies, Franck–Condon simulations of vibronic spectra using the double harmonic approximation, further analysis of the TGA simulated spectra presented in the main text, additional ms-TPES and discussion of contaminants in both the DPI and pyrolysis data, further analysis of the role of pyrolysis temperature, AIEs and VIEs used for the TGA simulations, justification for the choice of CBS-QB3 for AIEs, and alternative PES stationary points are described (PDF)
