# Supplementary material for: Threshold Photoelectron Spectroscopy, Dissociative Photoionization, and Pyrolysis of Aziridine
Source: J Phys Chem A. 2026 Jun 15;130(26):4839–49. doi: 10.1021/acs.jpca.5c08612 (PMC13339622; doi:10.1021/acs.jpca.5c08612)
Supplement: Supplementary file 1 [file jp5c08612_si_001.pdf]

Supporting Information to: Threshold Photoelectron Spectroscopy, Dissociative Photoionization, and Pyrolysis of Aziridine

Henry Cardwell,[†,‡] Domenik Schleier,[¶] Jens Petersen,[§] Michael Wenzel,[§] Rory McClish,[†,‡] Jerry Kamer,[‖] Patrick Hemberger,[⊥] Andras Bodi,[⊥] Roland Mitrić,[§] and Jordy Bouwman[*,†,‡,#]

†*Laboratory for Atmospheric and Space Physics, University of Colorado, Boulder, CO 80303, USA*

‡*Department of Chemistry, University of Colorado, Boulder, CO 80309, USA*

¶*Institut für Physik und Astronomie, Technische Universität Berlin, Hardenbergstr. 36, 10623 Berlin, Germany*

§*University of Würzburg, Institute of Physical and Theoretical Chemistry, Am Hubland, 97074 Würzburg, Germany*

‖*Laboratory for Astrophysics, Leiden Observatory, Leiden University, PO Box 9513, 2300 RA Leiden, The Netherlands*

⊥*Laboratory for Synchrotron Radiation and Femtochemistry, Paul Scherrer Institute, 5232 Villigen, Switzerland*

#*Institute for Modeling Plasma, Atmospheres, and Cosmic Dust (IMPACT), University of Colorado, Boulder, CO 80303, USA*

E-mail: jordy.bouwman@colorado.edu

Mass Selected Threshold Photoelectron Spectra

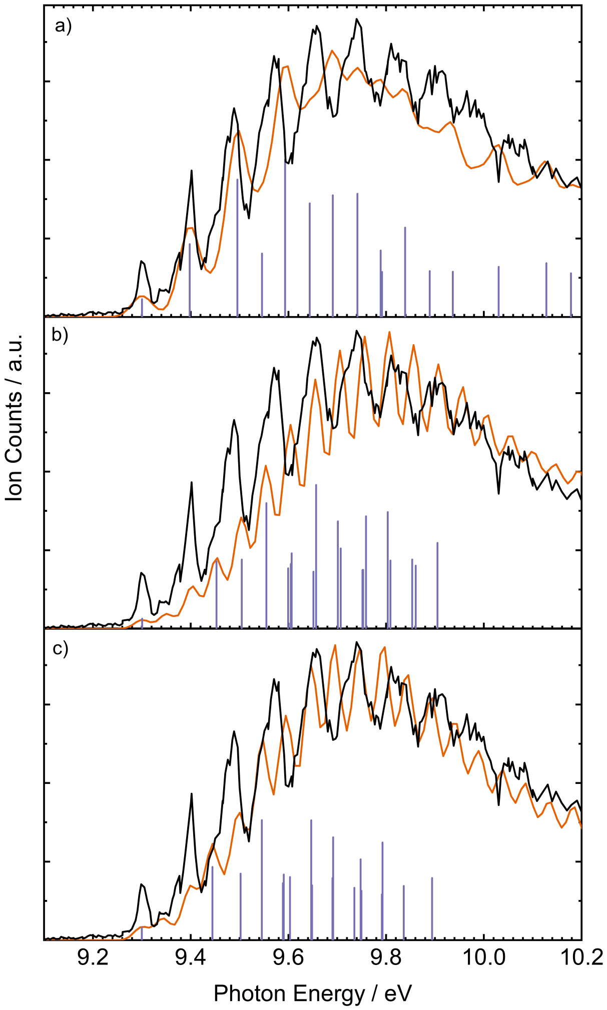

Figure S1: Franck–Condon simulation for the **AZR** ground-state photoelectron spectrum band in the double harmonic approximation using a) CCSD/aug-cc-pVTZ, b) M06-2X/aug-cc-pVTZ, and c) CCSD(T)/aug-cc-pVTZ. Black lines correspond to the experimental spectrum, purple lines represent the dominant Franck–Condon vibronic transitions. The full result is convolved with a Gaussian with a FWHM of 0.035 eV to obtain the orange traces. All simulations have been shifted to the AIE of 9.30 eV as determined using a).

In addition to the TGA results of the main text, double harmonic Franck–Condon simulations are shown in **Fig. S1**. The origin band in the CCSD spectrum was shifted to match the experimental spectrum to determine the AIE. The primary progression is due to ν_2 (see Fig. S3). A second progression of peaks grows in, which is attributed to the ν_9 mode. Overall, the first few transitions match quite well with the measured vibronic structure in the

CCSD simulation. However, there is a growing discrepancy between theory and experiment at higher photon energies. The CCSD(T) and M06-2X diverge even more from the experimental spectrum than the CCSD result, even at low energies. The method-dependence of the harmonic simulations as well as the quickly worsening agreement with increasing energy show that the harmonic approximation cannot adequately describe **AZR** photoionization at energies well above the origin region, while the TGA method captured the overall vibronic structure of the ground-state band well.

The wavepacket autocorrelation function of the **AZR** TGA simulation is presented in **Fig. S2** along with details regarding the contribution of various normal modes to the overall kinetic energy of the trajectory. After correlation is initially lost, only one recurrence with a notable intensity is observed after approximately 50 fs. This first recurrence, as seen in **Fig. S2a**, is already more than two orders of magnitude less than the initial correlation. While a second peak can be seen, it is about five orders of magnitude less intense than the original correlation.

A comparison to molecular vibrations can be obtained by projecting the nuclear coordinates along the trajectory on the harmonic normal modes. Inspecting the normal mode displacement and the contribution of each normal mode to the overall kinetic energy on the timescale of the autocorrelation function recurrences allows for the characterization of the nuclear motion as seen in **Fig. S2b**. In **Fig. S2c**, it can be seen that mode ν_2 is the primary contributor to the kinetic energy over the timescale of the first recurrence ($\sim$50 fs), with notable contributions from modes ν_1 and ν_9. The normal modes in question are illustrated in **Fig. S3**. Inspecting the time evolution of the angle between the N–H bond and the CCN plane as presented in **Fig. S2d** shows that the motion over this period occurs without crossing the barrier between the two double-well minima. In **Fig. S4**, the bond angle as well as the autocorrelation function are depicted over an extended time period, illustrating that the barrier is crossed eventually and the second potential well is reached (as indicated by the negative values of the angle between 400 and 500 fs). However, as the autocorrelation

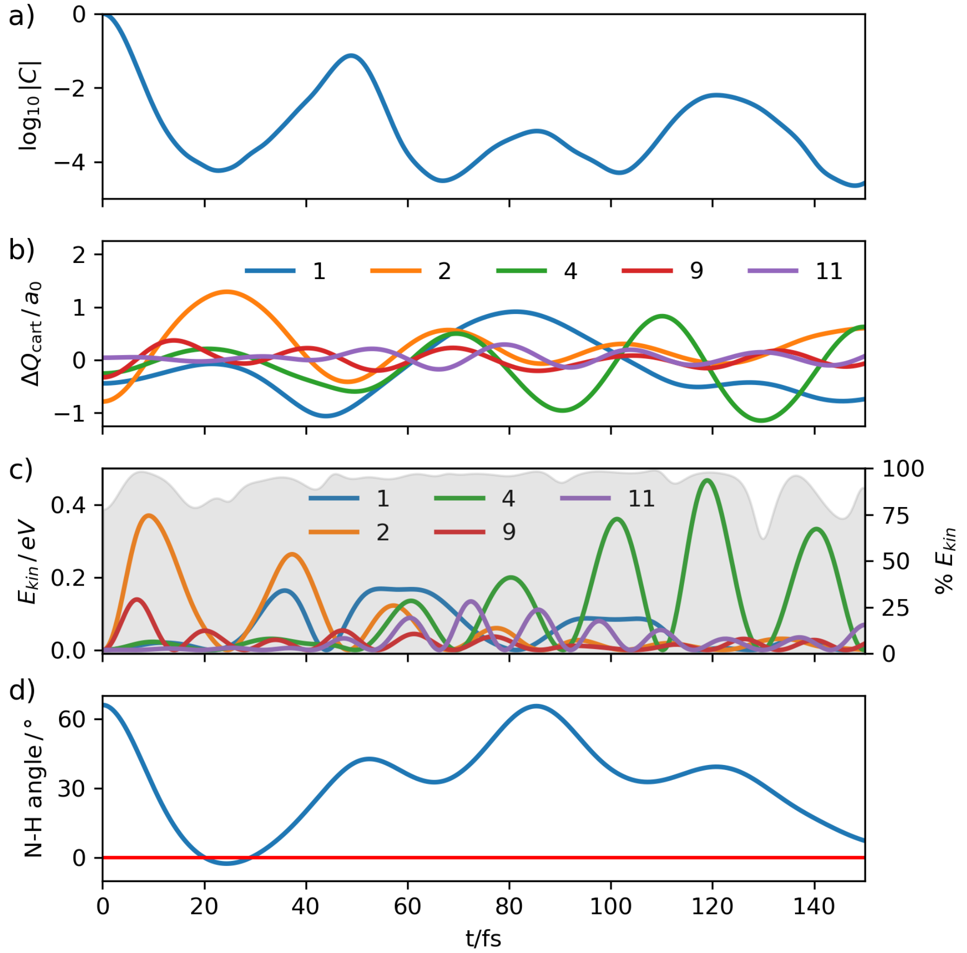

Figure S2: Analysis of the TGA simulation of aziridine at the ωB97X-D/aug-cc-pVTZ level of theory. a) Logarithm of the absolute value of the wavepacket autocorrelation function, b) Cartesian displacements of selected harmonic normal modes of the aziridine cation (for their character, see Fig. S3), c) Kinetic energy of the selected harmonic normal modes shown in b). The background area shaded in gray illustrates the percentage of the total kinetic energy contained in the selected normal modes (see right y-axis), d) Angle between the C–C–N ring plane and the N–H bond. Positive values correspond to the system being situated in one of the wells of the double well potential, while negative values pertain to the second well.

function becomes vanishingly small, the dynamics at these later times have no influence on the appearance of the spectrum. Overall, the vibronic progression is attributable to a combination of multiple modes that correspond to the N–H wagging motion. Due to anharmonic dynamics, the vibronic energy is quickly transferred to the other normal modes.

While M06-2X/aug-cc-pVTZ was used for the PES exploration, the TGA simulation was

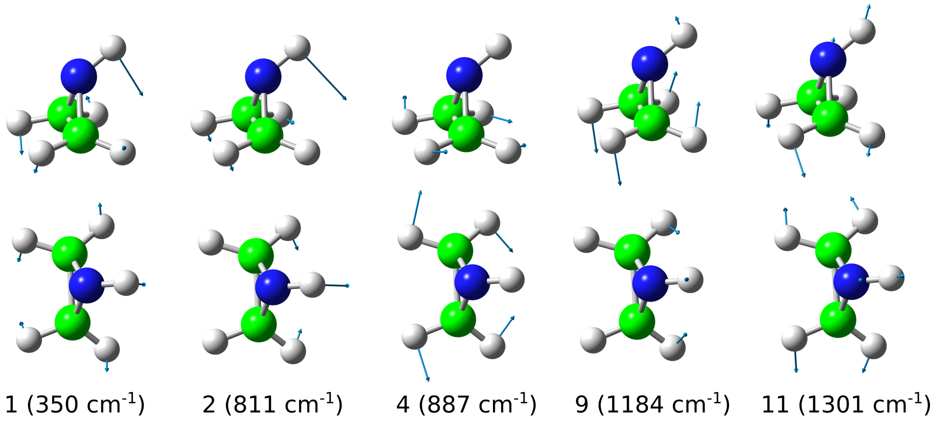

Figure S3: Illustration of the atomic displacements in the harmonic normal modes examined in Fig. S 2b.

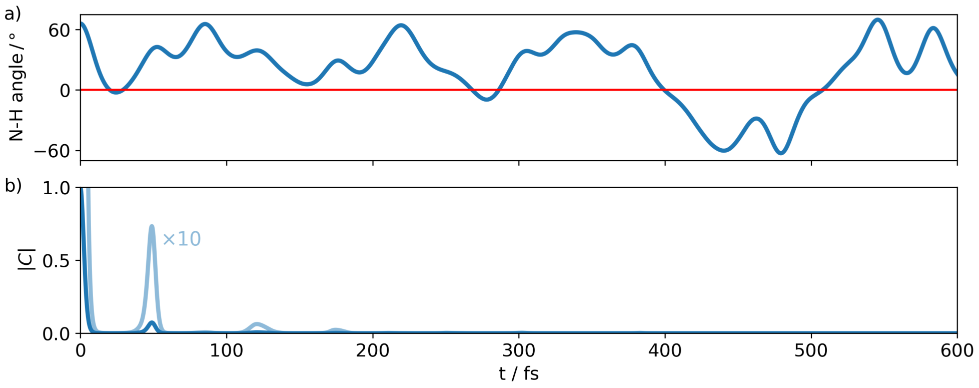

Figure S4: a) Angle between the C–C–N ring plane and the N–H bond along the TGA trajectory of aziridine cation. A value of 0° corresponds to the N–H bond being coplanar to the ring plane. b) Absolute value of the wavepacket autocorrelation function along the trajectory.

found to provide a better fit to the experimental data when using ωB97X-D/aug-cc-pVTZ, as seen in **Fig. S5**. As we are primarily trying to capture the most accurate trajectory physics, and thus the most accurate simulated spectrum, ωB97X-D/aug-cc-pVTZ was chosen for all following TGA spectra.

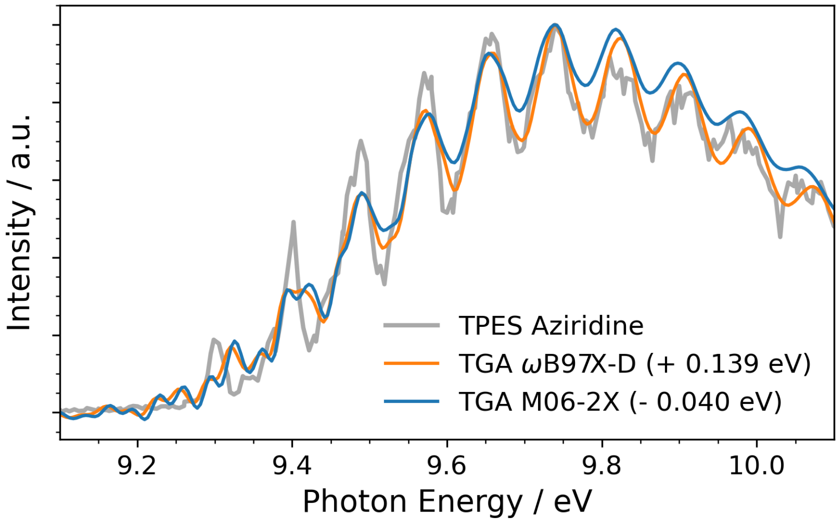

Figure S5: Comparison of TGA-simulated photoelectron spectra obtained using M06-2X/aug-cc-pVTZ (blue) and ωB97X-D/aug-cc-pVTZ (orange) to the experimental **AZR** spectrum (gray).

Dissociative Photoionization

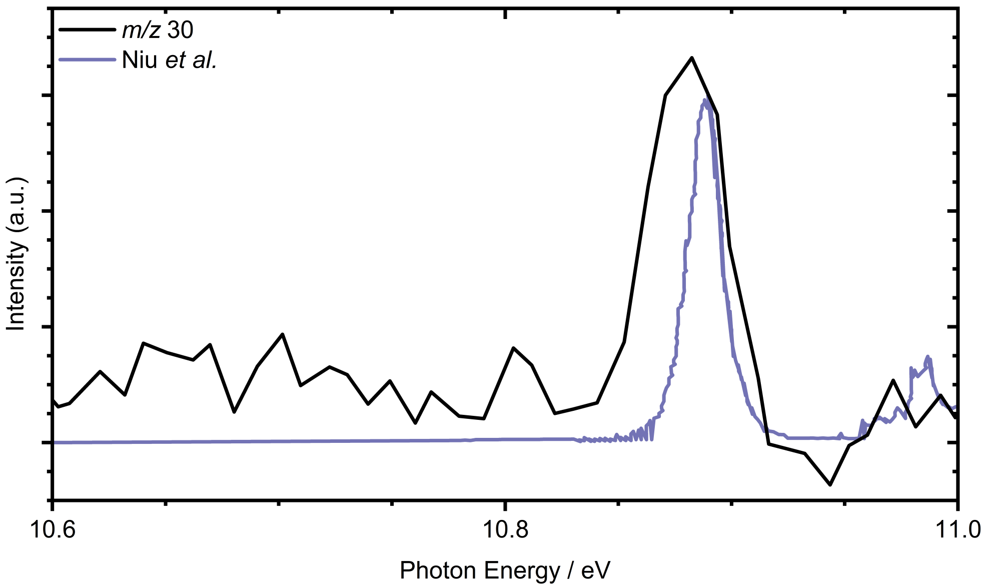

Figure S6: The ms-TPES of m/z 30 from 10.6 to 11.1 eV shown together with a literature threshold photoelectron spectrum of formaldehyde reproduced with permission.[1]

The cross-talk of formaldehyde contaminant in the ionization chamber (**Fig. S6**) at m/z 30 with the asymmetric peak of the m/z 28 fragment ion was subtracted by taking the average of two sections of the m/z 28 tail an equal distance from either side of the m/z 30 peak and subtracting that from the integrated intensity of the m/z 30 peak to obtain its contribution.

Pyrolysis

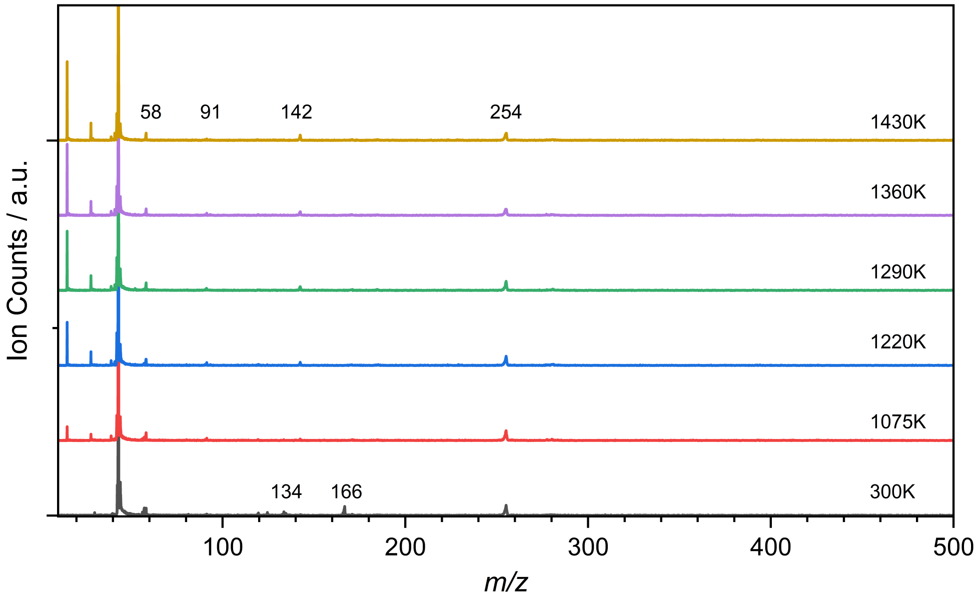

Figure S7: Mass spectra of **AZR** flowing through the SiC microreactor recorded at 10 eV and plotted from m/z 10 to 500 as a function of pyrolysis temperature. Trace acetone, C_7H_7, 4-vinylanisole, methyl iodide, propargyl iodide, and iodine (I_2) contaminants from previous experiments can be seen at m/z 58, 91, 134, 142, 166, and 254 respectively.

All-electron pyrolysate mass spectra at 10.0 eV photon energy are shown as a function of pyrolysis temperature in **Fig. S7**. m/z 43, 42, 28, and 15 are identified as **AZR** isomers and pyrolysis products with the carbon-13 peak of **AZR** evident at m/z 44. No new product peaks are found to grow in between pyrolysis temperatures of 1075 K and 1430 K. **Fig. S8** shows the fractional abundances of each pyrolysis product as a function of temperature. As m/z 15, the methyl cation peak is indicative of pyrolysis of **AZR** and its isomers, its comparable abundance with m/z 28 confirms that a significant fraction of this signal also

originates from pyrolysis and not solely from dissociative ionization.

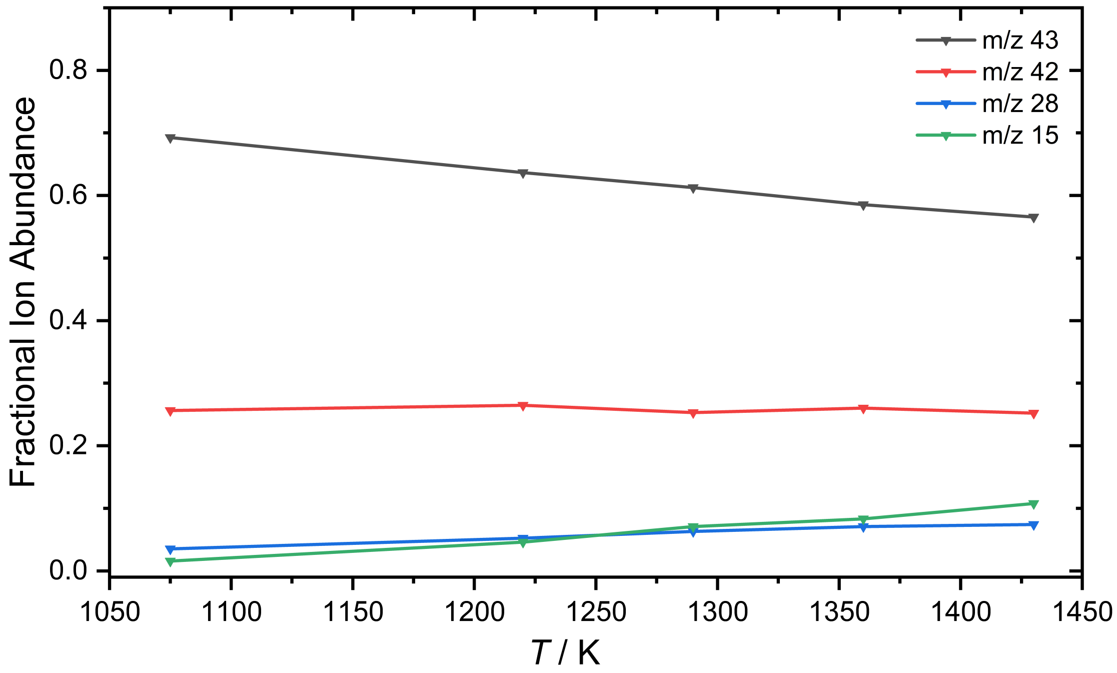

Figure S8: Fractional abundance of the pyrolysis products and the likely DPI peak at m/z 42 as a function of temperature.

The double-harmonic approximation simulation of the TPES of **EEN** is shown as a stick spectrum (blue) and as a convolved spectrum (orange) in **Fig. S9**, and can be seen to adequately capture the experimental vibronic structure. According to the harmonic simulation, the primary progression is due to the ν_4 mode of $\mathbf{EEN}^+$, which corresponds to the amine umbrella mode. The analysis of the trajectory underlying the TGA simulation, which is presented in **Fig. S10**, is consistent with this finding. The frequencies and characters of the normal modes dominating the dynamics are summarized in Table S1. The molecular structure inverts multiple times around the pyramidal NH_2 group. This is manifest in the regular oscillation of the displacement of mode ν_4 between positive and negative values (see **Fig. S10b**).

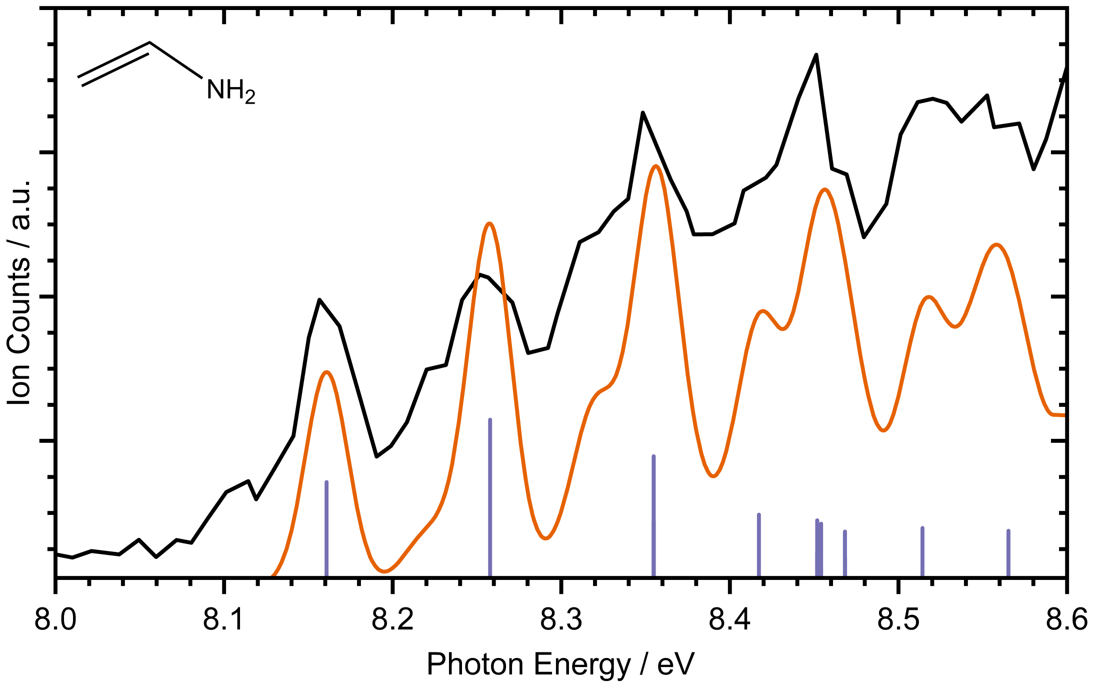

Figure S9: Pyrolysate ms-TPES of m/z 43 from 8.0 to 8.6 eV is shown in black and compared to Franck–Condon simulations of **EEN**. The stick spectrum (purple) is generated using the double harmonic approximation at the M06-2X/aug-cc-pVTZ level of theory. The convolution with a Gaussian of 0.03 eV FWHM (orange) accounts for the rotational envelope and the experimental resolution.

Table S1: Harmonic normal modes dominantly involved in the dynamics analyzed in Fig. S10.

Normal mode	Character	ν / $c \cdot \mathrm{cm}^{-1}$
4	H–N–H out-of-plane	783
9	C_α–H bending, C–C stretching	1293
12	C_α–H bending, C–C stretching	1584
13	NH_2 scissoring	1704
15	C_α–H stretching	3214

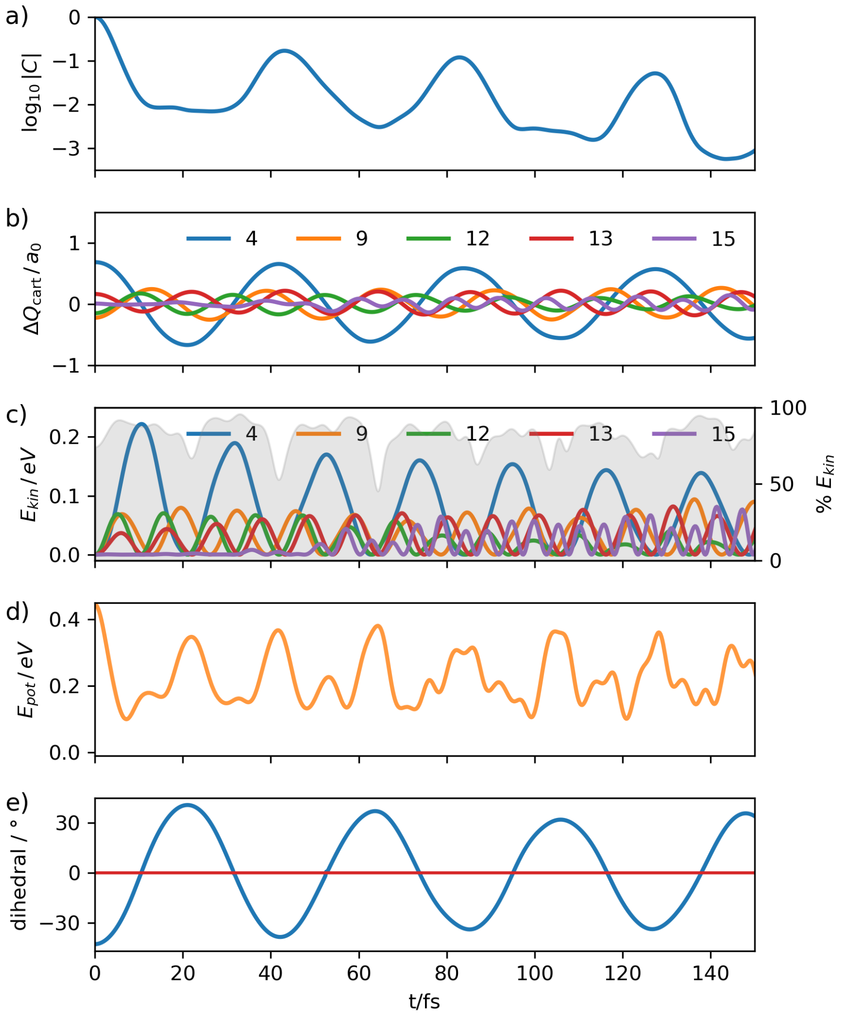

Figure S10: Analysis of the TGA simulation of ethenamine at the ωB97X-D/aug-cc-pVTZ level of theory. a) Logarithm of the absolute value of the wavepacket autocorrelation function, b) Cartesian displacements of selected harmonic normal modes of the ethenamine cation (for their character see Table S1), c) Kinetic energy of the selected harmonic normal modes shown in b). The background area shaded in gray illustrates the percentage of the total kinetic energy contained in the selected normal modes (see right y-axis), d) Electronic potential energy of the cation along the trajectory. The zero point of the energy corresponds to the optimized cation structure, e) Dihedral angle between the two planes formed by the NH_2 group and the atoms C_α, N, H, respectively. For a value of zero, both planes coincide, i.e, the group C_α-NH_2 is planar.

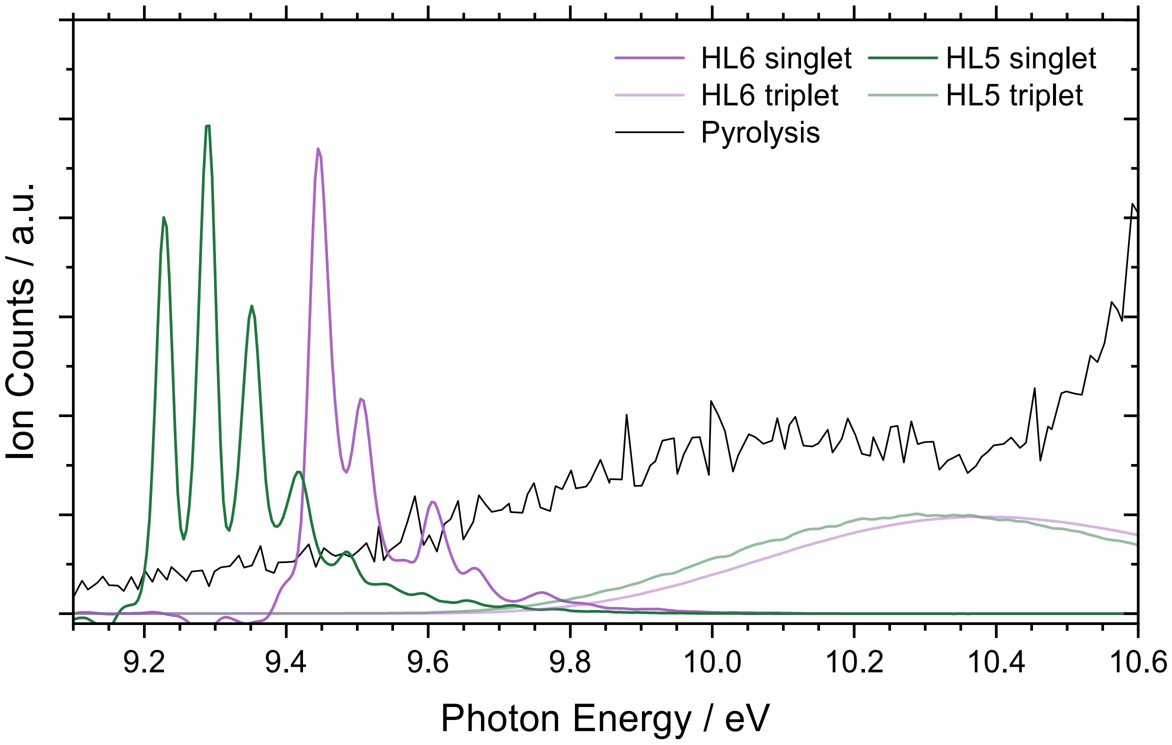

Figure S11: TGA simulated spectra of **HL5** and **HL6** into both their singlet and triplet cations. The experimental ms-TPES of m/z 42 is shown in black from 9.1 to 10.6 eV.

To further rule out the possibility of significant pyrolysis production of m/z 42, the vibronic spectra of the two lowest energy hydrogen loss isomers, **HL5** and **HL6**, were simulated using the TGA method (see Fig. S11). Ionization into both the singlet and triplet are shown, with the singlets being the ground cationic state for both molecules. The lower energy singlets can be shown to have strong origin transitions. The lack of a clear peak in the pyrolysis ms-TPES near the predicted maxima implies a low abundance of m/z 42 in the pyrolysate.

For TGA simulated spectra that could not be anchored to experimental data, a shift according to the difference between their ωB97X-D/aug-cc-pVTZ calculated vertical ionization energy (VIE) and the CBS-QB3 calculated VIE was applied. The VIEs were calculated using the electronic energies of the cation and the neutral at the optimized neutral geometry found with the corresponding level of theory. The relevant energies and the corresponding shift can

Table S2: VIEs for all isomers simulated with the TGA method from both CBS-QB3 and ωB97X-D/aug-cc-pVTZ

Molecule	VIE CBS-QB3 (eV)	VIE ωB97X-D (eV)	Difference (eV)
**AZR**	9.992	9.756	0.236
**EEN**	8.632	8.380	0.252
**MMA**	9.974	9.788	0.186
**E-EAN**	10.350	10.116	0.235
**Z-EAN**	10.300	10.079	0.222
**HL5** singlet	9.309	9.269	0.040
**HL5** triplet	10.446	10.157	0.289
**HL6** singlet	9.499	9.461	0.038
**HL6** triplet	10.505	10.227	0.278

be found in Table S2. For comparison, the calculated AIEs can be found in Table S3. AIEs are calculated including the harmonic ZPE of the neutral and cationic optimized structures at the corresponding level of theory.

Table S3: AIEs for all isomers simulated with the TGA method from both CBS-QB3 and ωB97X-D/aug-cc-pVTZ.

Molecule	AIE CBS-QB3 (eV)	AIE ωB97X-D (eV)	Difference (eV)
**AZR**	9.326	9.101	0.225
**EEN**	8.14	7.950	0.190
**MMA**	9.107	8.890	0.217
**E-EAN**	9.524	9.317	0.207
**Z-EAN**	9.504	9.301	0.203
**HL5** singlet	9.236	9.190	0.046
**HL5** triplet	9.648	9.389	0.259
**HL6** singlet	9.442	9.404	0.038
**HL6** triplet	9.655	9.402	0.253

Adiabatic ionization energy of aziridine using basis set extrapolation at the CCSD(T) level

The adiabatic ionization energy of aziridine was determined using CCSD(T) with a sequence of correlation-consistent basis sets ranging from cc-pVDZ through cc-pV5Z. The geometry and harmonic zero-point energies were calculated at the cc-pVQZ level of theory. All calculations were performed using the Gaussian 16 program package.[2] The absolute energies for the calculations used in the basis set extrapolation procedure are given in Table S4.

Table S4: Electronic energy contribution in Hartrees for aziridine at the CCSD(T)/cc-pVQZ-optimized geometries of the neutral or cation, respectively.

basis set	neutral E_{SCF}	neutral E_{corr}	cationic E_{SCF}	cationic E_{corr}
cc-pVDZ	-133.05028441	-0.49635645	-132.75306791	-0.46463097
cc-pVTZ	-133.08967474	-0.59981856	-132.79152615	-0.55857920
cc-pVQZ	-133.09903648	-0.63114456	-132.80029846	-0.58717236
cc-pV5Z	-133.10137418	-0.64147574	-132.80238176	-0.59649574
basis set limit	-133.10205240	-0.65231501	-132.80295996	-0.60627764

These energies were employed to obtain the basis set limit of the SCF and correlation energies separately. For the SCF energy, following Ref.,[3] an exponential fit of the form

$$E_{\mathrm{SCF}}(n) = E_{\mathrm{SCF},\infty} + b\,\mathrm{e}^{-cn} \tag{1}$$

was employed for the values obtained with all cc-pVnZ basis sets listed in Table S4. The extrapolated energies are listed in the last row of Table S4 in Hartrees. Fit parameters of $b = 0.902902\,E_H$ and $c = 1.42944$ were obtained for the neutral. Likewise, fit parameters of $b = 0.94784\,E_H$ and $c = 1.47218$ were obtained for the cation.

For the correlation energy, in line with Ref.,[4] only the values for the two largest basis sets cc-pVQZ and cc-pV5Z were used. Extrapolated energies were obtained using Equation 2:

$$E_{corr,\infty} = \frac{4^3\,E_{corr,QZ} - 5^3\,E_{corr,5Z}}{4^3 - 5^3} \tag{2}$$

These values are also listed in the last row of Table S4. The adiabatic ionization energy (without zero-point correction) is then obtained using Equation 3:

$$\Delta E_I = \left(E^{\text{cat}}_{SCF,\infty} + E^{\text{cat}}_{corr,\infty}\right) - \left(E^{\text{neut}}_{SCF,\infty} + E^{\text{neut}}_{corr,\infty}\right) = 9.39147\,\text{eV} \tag{3}$$

The harmonic zero-point corrections were obtained at the cc-pVQZ level of 0.0704068 E_H for the neutral and to 0.0682561 E_H for the cation. Including these values, a zero-point-corrected value for the adiabatic ionization energy is obtained as:

$$\Delta E_{I,ZP} = 9.33294\,\text{eV} \tag{4}$$

Given the fact that the harmonic approximation does not work well for the cation exhibiting a shallow double well potential, it is reasonable to assume that for this species the actual zero-point energy might be slightly smaller than the harmonic value. This reasoning, along with other small errors in the calculation, is in line with the fact that the experimentally observed value amounts to 9.30 eV, and matches with the CBS-QB3 AIE of 9.33 eV.

Potential Energy Surface Calculations

The energies of 42 and 28 amu neutrals and cationic species, including some not discussed in the main text, can be found in **Table S5**. The depicted cationic dissociation products include a singlet cation and a doublet radical neutral fragment of hydrogen or methyl. Triplet H_2CN^+ isomers have been discussed in the literature, and were found to be higher in energy than the singlets discussed in this work.[5] Isoenergetic H-loss isomers were not included in the above table.

Isomerization from **AZR** to **EEN** bypassing **EAN** was investigated on both the neutral and the cationic PES. Both pathways involved transferring a hydrogen from a carbon to the amine (**TS10**) to form **INT2**. Then, **INT2** ring opens through **TS11** to form **EEN**

Table S5: Hydrogen and methyl loss species with their corresponding abbreviations, neutral and cationic energies relative to the ground state of neutral AZR, and adiabatic ionization energies calculated using CBS-QB3.

Abbreviation	Description	Neutral Energy (eV)	DPI Energy (eV)	AIE
HL1	**MMA**: H loss from CH_2	3.82	9.87	6.05
HL2	**AZR**: H loss from CH_2	4.51	11.22	6.71
HL3	**E-EAN**: H loss from CH	3.18	9.39	6.21
HL4	**EEN**: H loss from CH	3.82	10.07	6.26
HL5	**E-EAN**: H loss from CH_3	3.02	12.25	9.24
HL6	**Z-EAN**: H loss from CH_3	3.04	12.48	9.44
HL7	**EAN**: H loss from NH	2.92	9.87	6.95
HL8	**AZR**: H loss from NH	3.24	11.90	8.65
HL9	**MMA**: H loss from CH_3	3.25	10.07	6.83
HL10	**Z-EAN**: H loss from CH	3.38	9.39	6.01
CH_3	HCNH + CH_3	2.93	9.90	N/A
CH_3	NCH_2 + CH_3	2.57	13.04	N/A

(**Fig. S12**). In both cases, the direct pathway was shown to be higher in energy than the pathway over **EAN** listed in the main text.

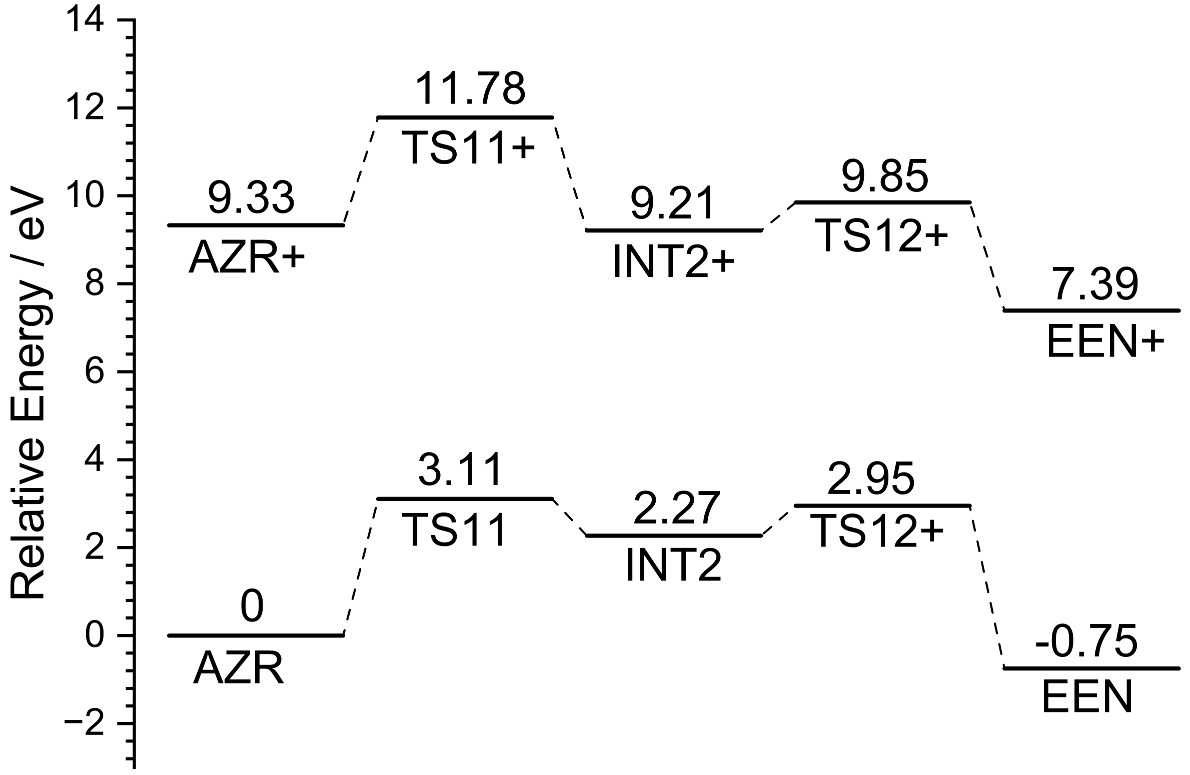

Figure S12: CBS-QB3 energetics of direct isomerization from **AZR** to **EEN** bypassing **EAN** isomers in the neutral and cation states.

Likewise, the possibility of open-shell species and triplet states of the carbene species was investigated. None of the stationary points described in the main text were found to have significant open-shell character. The triplet ground state for **1MAM** was found to be 1.94 eV higher in energy. Similarly, the triplet ground state for **1AE** was found to be 1.98 eV higher in energy than the singlet.

The stationary point structures and corresponding vibrational frequencies calculated using CBS-QB3 can be found below.

References

(1) Niu, B.; Shirley, D. A.; Bai, Y. High resolution photoelectron spectroscopy and femtosecond intramolecular dynamics of H2CO+ and D2CO+. *The Journal of Chemical Physics* **1993**, *98*, 4377–4390.

(2) Frisch, M. J.; Trucks, G. W.; Schlegel, H. B.; Scuseria, G. E.; Robb, M. A.; Cheeseman, J. R.; Scalmani, G.; Barone, V.; Petersson, G. A.; Nakatsuji, H.; Li, X; Caricato, M; Marenich, A. V.; Bloino, J.; Janesko, B. G.; Gomperts, R.; Mennucci, B.; Hratchian, H. P.; Ortiz, J. V.; Izmaylov, A. F.; Sonnenberg, J. L.; Williams-Young, D.; Ding, F.; Lipparini, F.; Egidi, F.; Goings, J; Peng, B.; Petrone, A.; Henderson, T.; Ranasinghe, D.; Zakrzewski, V. G.; Gao, J.; Rega, N.; Zheng, G.; Liang, W.; Hada, M.; Ehara, M.; Toyota, K.; Fukuda, R.; Hasegawa, J.; Ishida, M.; Nakajima, T.; Honda, Y.; Kitao, O.; Nakai, H.; Vreven, T.; Throssell, K.; Montgomery, Jr., J. A.; Peralta, J. E.; Ogliaro, F.; Bearpark, M. J.; Heyd, J. J.; Brothers, E. N.; Kudin, K. N.; Staroverov, V. N.; Keith, T. A.; Kobayashi, R.; Normand, J.; Raghavachari, K.; Rendell, A. P.; Burant, J. C.; Iyengar, S. S.; Tomasi, J.; Cossi, M.; Millam, J. M.; Klene, M.; Adamo, C.; Cammi, R.; Ochterski, J. W.; Martin, R. L.; Morokuma, K.; Farkas, O.; Foresman, J. B.; Fox, D. J. Gaussian 16 Revision C.02. 2019; Gaussian Inc. Wallingford CT.

(3) Helgaker, T.; Klopper, W.; Koch, H.; Noga, J. Basis-set convergence of correlated calculations on water. *The Journal of Chemical Physics* **1997**, *106*, 9639–9646.

(4) Halkier, A.; Helgaker, T.; Jørgensen, P.; Klopper, W.; Koch, H.; Olsen, J.; Wilson, A. K. Basis-set convergence in correlated calculations on Ne, N2, and H2O. *Chemical Physics Letters* **1998**, *286*, 243–252.

(5) Holzmeier, F.; Lang, M.; Hader, K.; Hemberger, P.; Fischer, I. H2CN+ and H2CNH+: New insight into the structure and dynamics from mass-selected threshold photoelectron spectra. *The Journal of Chemical Physics* **2013**, *138*, 214310.

Structures and Vibrational Wavenumbers of Potential Energy Surface Stationary Points from CBS-QB3 Calculations

AZR

C	-0.038329000000	-0.396780000000	0.742147000000
C	-0.038329000000	-0.396780000000	-0.742147000000
H	0.869578000000	-0.708546000000	1.250603000000
H	-0.953881000000	-0.608841000000	1.283692000000
N	-0.038329000000	0.874786000000	0.000000000000
H	0.896854000000	1.272637000000	0.000000000000
H	-0.953881000000	-0.608841000000	-1.283692000000
H	0.869578000000	-0.708546000000	-1.250603000000

Wavenumbers / cm^{-1}:

774.9084

860.5273

872.8775

918.3452

1005.5634

1108.059

1114.4131

1150.0943

1241.0449

1272.8787

1298.841

1498.6491

1527.9215

3094.3041

3100.0969

3172.3872

3186.1625

3499.3256

AZR+

C	-0.022112000000	-0.372403000000	0.798613000000
C	-0.022112000000	-0.372403000000	-0.798613000000
H	0.841814000000	-0.536171000000	1.435115000000
H	-0.983205000000	-0.733553000000	1.157871000000
N	-0.022112000000	0.785785000000	0.000000000000
H	0.702903000000	1.507794000000	0.000000000000
H	-0.983205000000	-0.733553000000	-1.157871000000
H	0.841814000000	-0.536171000000	-1.435115000000

Wavenumbers / cm^{-1}:

358.973
801.7317
868.7341
871.2117
927.713
1038.3306
1041.8068
1100.959
1175.0854
1271.717
1276.1716
1448.2845
1480.9785
3089.5118
3107.7223
3207.4478
3219.1853
3469.9037

MMA

C	1.147006000000	0.134757000000	-0.000007000000
N	-0.141413000000	-0.535562000000	0.000078000000
C	-1.179518000000	0.182510000000	-0.000063000000
H	1.713727000000	-0.188213000000	-0.878187000000
H	1.713597000000	-0.187736000000	0.878437000000
H	1.077350000000	1.233779000000	-0.000301000000
H	-1.160753000000	1.283408000000	-0.000229000000
H	-2.158958000000	-0.295905000000	0.000154000000

Wavenumbers / cm^{-1}:

222.2715

480.7028

700.802

964.6967

1061.4651

1123.3861

1142.6764

1238.3582

1435.7867

1476.3953

1479.2683

1508.4741

1738.8194

2957.0219

2967.7254

3060.7477

3079.5001

3118.8913

MMA+

C	1.271722000000	0.047849000000	0.000048000000
N	-0.117277000000	-0.207015000000	-0.000009000000
C	-1.321452000000	0.066254000000	-0.000062000000
H	1.717893000000	-0.378630000000	-0.902055000000
H	1.717637000000	-0.377582000000	0.902767000000
H	1.418785000000	1.142234000000	-0.000609000000
H	-1.664265000000	1.112925000000	-0.000173000000
H	-2.070737000000	-0.734460000000	0.000218000000

Wavenumbers / cm^{-1}:

130.6968
228.2941
348.8614
875.9624
972.4472
1032.1349
1105.8195
1120.7448
1382.8189
1389.3872
1400.1252
1449.1044
1822.7494
2943.6402
2970.545
3072.5014
3079.0998
3124.2379

EEN

C	-1.251519000000	-0.195712000000	0.016107000000
C	-0.068792000000	0.426390000000	-0.001040000000
H	-1.333824000000	-1.277756000000	0.022485000000
H	-2.169863000000	0.374318000000	0.001178000000
N	1.186507000000	-0.168256000000	-0.085093000000
H	-0.029551000000	1.512480000000	-0.012130000000
H	1.213074000000	-1.154672000000	0.133570000000
H	1.936479000000	0.339348000000	0.360150000000

Wavenumbers / cm^{-1}:

358.352
470.211
593.9682
709.4224
811.0318
963.292
1001.8209
1077.6748
1289.1538
1337.8472
1452.2307
1653.533
1714.0529
3136.9606
3146.4356
3232.9391
3552.129
3652.7948

EEN+

C	-1.257818000000	-0.191695000000	0.000027000000
C	-0.015529000000	0.455693000000	-0.000006000000
H	-1.336266000000	-1.273852000000	-0.000774000000
H	-2.168696000000	0.392985000000	0.000610000000
N	1.130009000000	-0.191548000000	0.000081000000
H	0.042487000000	1.539645000000	-0.000536000000
H	1.176441000000	-1.207575000000	0.000161000000
H	2.016048000000	0.305646000000	-0.000158000000

Wavenumbers / cm^{-1}:

453.4641
460.5453
602.4574
770.3763
916.9827
980.1777
1046.5633
1067.9307
1283.0623
1366.7715
1479.0258
1560.8388
1690.6649
3150.228
3191.2332
3266.1875
3499.5462
3607.9868

Z-EAN

C	1.018112000000	-0.628278000000	0.000000000000
C	0.000000000000	0.480772000000	0.000000000000
H	1.666901000000	-0.550200000000	0.879007000000
H	1.666901000000	-0.550200000000	-0.879007000000
H	0.540923000000	-1.611574000000	0.000000000000
N	-1.264293000000	0.385931000000	0.000000000000
H	0.414254000000	1.495274000000	0.000000000000
H	-1.547597000000	-0.599786000000	0.000000000000

Wavenumbers / cm^{-1}:

171.8215
488.2828
692.2728
902.5465
1064.6068
1075.5361
1148.9601
1285.0656
1394.5455
1431.5976
1472.5399
1478.1357
1727.4945
3015.28
3045.5325
3071.1336
3100.0271
3394.5503

Z-EAN+

C	1.047729000000	-0.607394000000	0.000000000000
C	0.000000000000	0.482583000000	0.000000000000
H	1.669924000000	-0.448849000000	0.887446000000
H	1.669924000000	-0.448849000000	-0.887446000000
H	0.612906000000	-1.604025000000	0.000000000000
N	-1.222955000000	0.294738000000	0.000000000000
H	0.341201000000	1.529265000000	0.000000000000
H	-2.019641000000	-0.341843000000	0.000000000000

Wavenumbers / cm^{-1}:

180.1851
366.4207
503.5873
681.8841
796.621
873.5898
1054.3404
1068.356
1250.2401
1347.9045
1445.0124
1448.4772
1682.5307
3007.1837
3029.4247
3096.0897
3172.2553
3540.434

E-EAN

6	1.030540000	-0.639276000	0.000000000
6	0.000000000	0.449807000	0.000000000
1	1.677955000	-0.555058000	0.879706000
1	1.677955000	-0.555058000	-0.879706000
1	0.542603000	-1.614274000	0.000000000
7	-1.241663000	0.190335000	0.000000000
1	0.399013000	1.475538000	0.000000000
1	-1.789124000	1.053318000	0.000000000

Wavenumbers / cm^{-1}:

196.6304
490.9973
684.2263
921.4895
1060.6483
1077.8366
1131.1554
1277.2074
1389.0288
1438.4316
1471.2633
1478.6019
1729.9372
2977.7229
3019.6999
3068.1045
3125.9136
3441.9038

E-EAN+

C	1.064119000000	-0.626316000000	0.000000000000
C	0.000000000000	0.427616000000	0.000000000000
H	1.691450000000	-0.462351000000	0.882791000000
H	1.691450000000	-0.462351000000	-0.882791000000
H	0.642001000000	-1.628304000000	0.000000000000
N	-1.222702000000	0.235351000000	0.000000000000
H	0.320907000000	1.489244000000	0.000000000000
H	-2.171606000000	0.608506000000	0.000000000000

Wavenumbers / cm^{-1}:

170.5327
386.9234
479.4125
670.087
760.7452
897.1101
1056.7632
1080.7612
1229.7139
1356.8537
1447.1105
1454.6188
1686.2077
2907.8771
3031.8828
3093.3197
3173.8072
3542.4523

1MAM

C	-1.127861000000	0.172886000000	0.000000000000
N	0.214767000000	-0.411752000000	0.000000000000
C	1.376150000000	0.173156000000	-0.000001000000
H	-1.677266000000	-0.142450000000	0.886306000000
H	-1.677268000000	-0.142455000000	-0.886302000000
H	-1.027778000000	1.254687000000	-0.000003000000
H	1.172693000000	1.261657000000	0.000001000000
H	0.216516000000	-1.425433000000	-0.000001000000

Wavenumbers / cm^{-1}:

165.4129

509.133

600.4649

921.3707

1089.2072

1113.6774

1137.427

1305.803

1419.0007

1478.655

1484.8389

1493.703

1557.4117

2867.8975

3023.9785

3087.9436

3133.1232

3488.8346

MAM+

C	-1.134614000000	0.202877000000	0.000000000000
N	0.213141000000	-0.429392000000	0.000001000000
C	1.308436000000	0.166782000000	-0.000001000000
H	-1.654681000000	-0.131723000000	0.893950000000
H	-1.654681000000	-0.131724000000	-0.893948000000
H	-1.011444000000	1.280784000000	-0.000001000000
H	1.556537000000	1.224095000000	-0.000003000000
H	0.229353000000	-1.453643000000	0.000001000000

Wavenumbers / cm^{-1}:

175.0551
421.8844
674.0463
850.1863
955.6675
958.2184
1124.8992
1166.858
1411.413
1429.1272
1469.4057
1484.0797
1732.9601
3064.2962
3157.0223
3181.5583
3184.9387
3411.8752

1AE

C	1.149248000000	-0.319893000000	0.000000000000
C	0.000000000000	0.642642000000	0.000000000000
H	1.779679000000	-0.114204000000	0.871380000000
H	1.779679000000	-0.114204000000	-0.871380000000
H	0.877881000000	-1.394031000000	0.000000000000
N	-1.150871000000	0.020741000000	0.000000000000
H	-1.260339000000	-1.003229000000	0.000000000000
H	-2.016291000000	0.543990000000	0.000000000000

Wavenumbers / cm^{-1}:

181.6447

484.164

709.5537

754.7544

927.677

1029.5053

1038.4163

1236.8569

1378.9654

1430.165

1450.1227

1496.8775

1671.3268

2887.5067

3054.4364

3076.6954

3261.9659

3581.5818

A E+

C	1.246826000000	-0.332640000000	0.000000000000
C	0.000000000000	0.393937000000	0.000000000000
H	1.830712000000	-0.038146000000	0.880789000000
H	1.830712000000	-0.038146000000	-0.880789000000
H	1.092315000000	-1.420796000000	0.000000000000
N	-1.240772000000	0.147521000000	0.000000000000
H	-1.620679000000	-0.806777000000	0.000000000000
H	-1.928613000000	0.903434000000	0.000000000000

Wavenumbers / cm^{-1}:

155.1268

361.3003

537.4143

826.6838

917.8296

972.1736

1016.8368

1203.3311

1374.47

1397.4733

1418.9519

1598.6284

1761.8045

2984.6564

3068.3005

3074.0869

3366.8167

3493.8802

INT1

C	-0.000006000000	-0.184428000000	1.225666000000
C	-0.000006000000	-0.184428000000	-1.225666000000
H	0.000019000000	0.484393000000	2.068238000000
H	0.000057000000	-1.256619000000	1.337981000000
N	-0.000006000000	0.342823000000	0.000000000000
H	-0.000041000000	1.357827000000	0.000000000000
H	0.000057000000	-1.256619000000	-1.337981000000
H	0.000019000000	0.484393000000	-2.068238000000

Wavenumbers / cm^{-1}:

347.7151
477.8282
480.3865
543.0536
673.5055
960.1209
1048.2143
1101.6301
1306.4182
1430.6641
1464.7916
1516.6851
1642.3309
3184.4996
3191.4642
3310.5268
3310.9786
3546.9286

INT1+

C	-0.000009000000	-0.201722000000	1.197151000000
C	-0.000009000000	-0.201722000000	-1.197151000000
H	0.000053000000	0.420169000000	2.080948000000
H	0.000017000000	-1.282558000000	1.250927000000
N	-0.000009000000	0.390480000000	0.000000000000
H	0.000028000000	1.412080000000	0.000000000000
H	0.000017000000	-1.282558000000	-1.250927000000
H	0.000053000000	0.420169000000	-2.080948000000

Wavenumbers / cm^{-1}:

486.1546

524.5446

556.7171

872.9924

888.3834

1010.6937

1050.2335

1110.2038

1225.4275

1332.4679

1476.7882

1535.7305

1581.5975

3156.4679

3168.2376

3300.1342

3301.1269

3486.966

INT2

C	0.299146000000	0.922086000000	-0.199646000000
C	0.658897000000	-0.508642000000	0.019899000000
H	-1.264264000000	-0.297446000000	0.886682000000
H	0.338469000000	1.441261000000	0.761240000000
N	-0.776345000000	-0.168490000000	0.010748000000
H	-1.326616000000	-0.412440000000	-0.798217000000
H	0.965775000000	-0.921276000000	0.978481000000
H	0.972796000000	-1.111333000000	-0.824937000000

Wavenumbers / cm^{-1}:

534.942

725.762

814.0821

858.4751

899.9691

958.3395

1101.0363

1160.4022

1168.1636

1241.1438

1264.3313

1521.3119

1633.6611

2986.756

3018.5902

3093.7967

3485.9748

3608.8866

INT2+

C	0.025683000000	0.882788000000	-0.163917000000
C	0.800242000000	-0.315807000000	0.028821000000
H	-1.193437000000	-0.534088000000	0.897379000000
H	-0.161426000000	1.785694000000	0.393681000000
N	-0.704818000000	-0.363748000000	0.020450000000
H	-1.162947000000	-0.774564000000	-0.791252000000
H	1.244109000000	-0.529039000000	0.994191000000
H	1.251881000000	-0.803650000000	-0.826580000000

Wavenumbers / cm^{-1}:

611.5336
732.8056
819.4958
874.4248
917.9855
1024.0145
1082.9667
1117.2879
1156.5991
1187.3938
1298.7517
1465.2915
1630.8741
3100.8116
3194.044
3244.4323
3434.8146
3529.9342

TS1

C	1.021535000000	-0.287486000000	0.045597000000
C	-1.021536000000	-0.287486000000	-0.045598000000
H	1.809778000000	-0.296934000000	-0.707356000000
H	1.108278000000	-0.938766000000	0.902912000000
N	0.000000000000	0.613581000000	0.000003000000
H	0.000002000000	1.626164000000	-0.000010000000
H	-1.108275000000	-0.938765000000	-0.902914000000
H	-1.809781000000	-0.296934000000	0.707352000000

Wavenumbers / cm^{-1}:

-619.9476

78.3394

644.4061

728.2015

806.0948

808.245

1132.3084

1142.5025

1260.5228

1328.7791

1472.6813

1501.3156

1543.5438

3064.9559

3074.1344

3212.4961

3216.6484

3490.6093

TS1+

C	-1.052941000000	-0.223758000000	0.083299000000
H	-1.536208000000	-0.066929000000	1.038742000000
H	-1.402506000000	-0.930800000000	-0.657909000000
N	0.070342000000	0.595951000000	-0.243967000000
C	0.980071000000	-0.327148000000	0.062111000000
H	0.939115000000	-1.287936000000	-0.441934000000
H	1.798215000000	-0.114763000000	0.749457000000
H	0.146216000000	1.534204000000	0.146958000000

Wavenumbers / cm^{-1}:

-729.0763

530.6809

659.2139

718.6263

876.5974

992.7884

1058.7799

1088.9683

1163.6554

1340.625

1436.0909

1447.5059

1544.7686

3101.5227

3141.0287

3229.8571

3294.6312

3507.7499

TS2

C	1.206493000000	0.183911000000	0.036738000000
N	-0.053866000000	-0.395653000000	-0.104932000000
C	-1.224950000000	0.157568000000	-0.002878000000
H	2.011700000000	-0.403958000000	-0.383674000000
H	0.588488000000	-0.902813000000	0.727960000000
H	1.323098000000	1.265942000000	0.110502000000
H	-1.336761000000	1.239925000000	0.041048000000
H	-2.098723000000	-0.478397000000	0.035527000000

Wavenumbers / cm^{-1}:

-1688.2441
416.4779
582.4749
672.1862
771.2395
799.6448
1063.1923
1095.3418
1198.0344
1259.9231
1469.8426
1491.094
1598.9028
2342.2427
3065.1615
3086.0801
3218.5374
3221.2423

TS2+

6	1.226918000	0.159553000	0.018060000
7	-0.059956000	-0.313364000	-0.109896000
6	-1.258925000	0.128919000	0.002871000
1	1.962815000	-0.284695000	-0.645321000
1	0.751606000	-0.932842000	0.659688000
1	1.429981000	1.063682000	0.588841000
1	-1.477807000	1.167301000	-0.252006000
1	-2.054860000	-0.550726000	0.292485000

Wavenumbers / cm^{-1}:

-1914.0879

289.0874

438.4163

666.8621

954.0035

1017.6554

1047.485

1065.0324

1114.7185

1177.493

1452.2922

1472.9223

1614.4547

2129.613

3079.6699

3093.8637

3217.9434

3237.2293

TS3

C	1.110038000000	-0.371215000000	-0.000001000000
C	-1.139634000000	-0.247303000000	0.000000000000
H	2.143436000000	0.018771000000	-0.000001000000
H	0.114872000000	-1.262974000000	-0.000001000000
N	0.150684000000	0.488218000000	0.000001000000
H	0.291063000000	1.499695000000	0.000002000000
H	-1.713291000000	0.019044000000	0.894942000000
H	-1.713291000000	0.019046000000	-0.894942000000

Wavenumbers / cm^{-1}:

-1576.5634

93.2207

720.8299

769.3812

897.4325

951.2386

1009.1779

1139.2245

1175.3597

1218.8274

1385.9184

1453.6406

1632.9079

1999.9638

2956.3605

3010.3071

3074.19

3451.3522

TS3+

C	1.013442000000	-0.401368000000	-0.000008000000
C	-1.079689000000	-0.190813000000	0.000000000000
H	2.106582000000	-0.386868000000	-0.000011000000
H	-0.136291000000	-1.235437000000	-0.000017000000
N	0.187793000000	0.563574000000	0.000008000000
H	0.353548000000	1.572423000000	0.000021000000
H	-1.620459000000	-0.171014000000	-0.941873000000
H	-1.620455000000	-0.171040000000	0.941877000000

Wavenumbers / cm^{-1}:

-2108.6719
473.3658
694.7694
730.2398
947.7214
949.5632
1024.5131
1095.948
1124.3475
1179.8947
1338.2591
1425.347
1691.4864
1942.586
3103.3612
3114.1849
3240.9964
3481.6579

TS4+

C	-1.300738000000	0.064991000000	-0.000003000000
N	0.119146000000	-0.122809000000	0.000003000000
C	1.263694000000	-0.174179000000	0.000006000000
H	-1.703435000000	-0.406645000000	0.897718000000
H	-1.703433000000	-0.406694000000	-0.897700000000
H	-1.505176000000	1.137653000000	-0.000032000000
H	2.013432000000	1.701556000000	-0.000044000000
H	2.286853000000	-0.511080000000	0.000016000000

Wavenumbers / cm^{-1}:

-621.1256

54.618

245.2739

343.0005

469.8818

766.3739

845.3105

881.8076

1130.1138

1136.355

1432.2087

1456.8769

1457.9381

2265.2153

3048.8452

3140.6292

3147.5373

3346.2048

TS5+

C	1.747363000000	-0.179955000000	0.000001000000
N	-0.649742000000	0.470277000000	0.000000000000
C	-1.507231000000	-0.304347000000	-0.000001000000
H	1.990643000000	0.309495000000	0.935183000000
H	1.990644000000	0.309497000000	-0.935181000000
H	1.588026000000	-1.250471000000	-0.000001000000
H	-2.116018000000	-1.192975000000	-0.000002000000
H	-0.345888000000	1.438332000000	0.000001000000

Wavenumbers / cm^{-1}:

-270.6159
25.7959
223.8958
397.2813
425.1888
464.3076
603.1745
667.9867
853.3565
923.7113
1408.0611
1412.0781
2063.1673
3087.4264
3267.9817
3272.0002
3333.6441
3601.3356

TS6

C	-0.086929000000	0.503052000000	-0.058153000000
C	1.203540000000	-0.232242000000	-0.103609000000
H	-0.163621000000	1.382802000000	-0.719896000000
N	-1.146956000000	-0.359126000000	0.155641000000
H	-1.858524000000	-0.214605000000	-0.568559000000
H	1.154890000000	-1.302708000000	-0.278501000000
H	0.143497000000	0.897227000000	0.973083000000
H	2.052786000000	0.126308000000	0.474958000000

Wavenumbers / cm^{-1}:

-607.6838

461.4389

696.0299

724.3965

792.6949

1005.3529

1026.936

1092.4574

1208.4245

1213.5883

1301.3674

1393.2009

1475.8172

2756.9553

2930.7006

3098.3835

3215.9148

3397.8474

TS6+

C	-1.130702000000	-0.255630000000	-0.034196000000
C	0.056854000000	0.570209000000	0.072579000000
H	-1.989695000000	-0.003291000000	-0.660088000000
H	-1.127209000000	-1.212441000000	0.488498000000
N	1.080203000000	-0.436243000000	0.002538000000
H	0.094196000000	1.409524000000	-0.635654000000
H	0.018811000000	1.055157000000	1.089147000000
H	1.885562000000	-0.082726000000	-0.529965000000

Wavenumbers / cm^{-1}:

-535.6492
250.0389
513.8627
616.6001
822.1975
930.4914
979.1385
1080.7787
1213.4806
1263.7327
1316.202
1368.9427
1488.1455
2731.3596
3016.046
3094.5457
3217.5064
3407.9473

TS7+

C	0.444772000000	-1.197622000000	0.000000000000
C	0.000000000000	0.187385000000	0.000000000000
H	1.045992000000	-1.392649000000	0.891939000000
H	1.045992000000	-1.392649000000	-0.891939000000
H	-0.448842000000	-1.831558000000	0.000000000000
N	-0.701597000000	1.105265000000	0.000000000000
H	1.723035000000	0.917658000000	0.000000000000
H	-1.123631000000	2.023769000000	0.000000000000

Wavenumbers / cm^{-1}:

-778.0124
179.8438
440.5922
447.2228
496.8145
577.7089
594.0414
903.6015
1035.7814
1052.91
1387.8329
1428.1533
1437.2048
2249.4524
3027.3739
3111.4339
3120.6383
3672.6177

TS8

C	1.140578000000	-0.195783000000	-0.031730000000
C	-0.076592000000	0.554146000000	0.027234000000
H	1.356027000000	-0.696785000000	0.912793000000
H	2.032735000000	0.223441000000	-0.492915000000
H	-0.079909000000	-1.133105000000	-0.245892000000
N	-1.066322000000	-0.300687000000	-0.080861000000
H	-0.237833000000	1.630384000000	0.116818000000
H	-1.990678000000	-0.069304000000	0.302199000000

Wavenumbers / cm^{-1}:

-1997.1842

537.2164

695.2173

773.3039

802.6414

966.0446

1069.833

1081.5606

1149.9082

1205.8017

1401.0362

1470.3972

1558.6201

1882.8942

3078.2255

3099.4599

3146.8123

3358.9112

TS8+

C	1.108156000000	-0.150533000000	-0.000003000000
C	-0.164882000000	0.627117000000	-0.000001000000
H	1.654387000000	-0.228938000000	0.939723000000
H	1.654380000000	-0.228944000000	-0.939733000000
H	0.193271000000	-1.177114000000	0.000002000000
N	-0.967829000000	-0.352249000000	0.000004000000
H	-0.397164000000	1.688856000000	-0.000002000000
H	-1.989718000000	-0.447620000000	0.000009000000

Wavenumbers / cm^{-1}:

-1846.2501
425.5258
596.2792
716.5872
921.9899
965.5298
994.3952
1054.1142
1084.6097
1132.0248
1296.3284
1384.291
1687.5894
1868.5765
3078.7993
3189.1638
3196.8876
3429.9473

TS9

6	1.212353000	-0.193629000	0.000000000
6	-0.171917000	0.429962000	-0.000008000
1	1.774131000	0.142368000	0.879349000
1	1.774104000	0.142243000	-0.879413000
1	1.145999000	-1.282169000	0.000087000
7	-1.243614000	-0.183179000	-0.000004000
1	-0.134966000	1.543962000	0.000027000
1	-2.096585000	-0.682145000	0.000023000

Wavenumbers / cm^{-1}:

-1171.4576
153.3623
500.7917
610.983
774.1499
873.1917
1093.9554
1100.0905
1374.342
1425.6749
1468.4432
1474.1772
1816.9809
2808.2188
3009.2166
3062.3784
3121.0911
3944.8306

TS9+

C	-1.211335000000	-0.196459000000	0.000006000000
C	0.160088000000	0.426246000000	0.000013000000
H	-1.737067000000	0.179167000000	-0.884415000000
H	-1.737035000000	0.179080000000	0.884484000000
H	-1.169307000000	-1.282629000000	-0.000047000000
N	1.231420000000	-0.184776000000	-0.000020000000
H	0.220589000000	1.530770000000	0.000054000000
H	2.110364000000	-0.691678000000	-0.000053000000

Wavenumbers / cm^{-1}:

-493.4972

171.2271

421.1161

423.5019

772.0007

878.2178

1051.2638

1056.206

1242.4706

1352.9243

1448.7358

1450.6328

1684.2058

2939.7662

3030.8969

3094.964

3176.2687

3618.5533

TS10

C	-1.196507000000	0.196347000000	-0.018417000000
C	-0.005304000000	-0.521555000000	-0.132313000000
H	-0.778526000000	-0.751573000000	0.865287000000
H	-2.130982000000	-0.314560000000	-0.222618000000
H	-1.260534000000	1.290771000000	0.095094000000
N	1.160133000000	0.135292000000	0.021024000000
H	1.232115000000	1.149788000000	0.034535000000
H	2.027860000000	-0.370223000000	-0.015086000000

Wavenumbers / cm^{-1}:

-1476.608
348.6696
450.1072
634.914
697.5793
929.645
1038.7242
1103.0069
1237.5007
1331.7417
1454.3633
1529.1726
1661.0365
2141.1278
2952.2776
3180.332
3450.0364
3674.6542

TS10+

C	-1.276418000000	0.160536000000	-0.010805000000
C	0.002426000000	-0.339531000000	-0.131981000000
H	-0.747300000000	-0.904612000000	0.730983000000
H	-2.094373000000	-0.379016000000	-0.480564000000
H	-1.492344000000	1.094077000000	0.514570000000
N	1.218355000000	0.103979000000	0.012991000000
H	1.465213000000	1.064144000000	-0.220620000000
H	1.984275000000	-0.528482000000	0.221409000000

Wavenumbers / cm^{-1}:

-1503.3983

381.8103

523.0136

621.2618

695.2289

972.1918

1009.2332

1072.1266

1179.6919

1242.4785

1440.0789

1618.227

1690.8331

2172.2493

3068.8275

3203.5473

3475.0844

3594.9075

TS11

C	-0.950384000000	-0.436951000000	-0.210118000000
C	-0.091277000000	0.772946000000	-0.002390000000
H	0.158585000000	-1.240839000000	-0.150663000000
H	-1.295194000000	-0.706841000000	0.805216000000
N	0.871971000000	-0.347215000000	0.183610000000
H	1.381539000000	-0.390284000000	-0.705348000000
H	-0.204840000000	1.352429000000	0.910898000000
H	0.106079000000	1.400070000000	-0.870327000000

Wavenumbers / cm^{-1}:

-1085.6205
613.1035
717.2777
854.7011
987.3593
1058.4437
1121.4414
1161.7444
1224.7854
1273.4357
1306.3838
1538.8275
1649.3817
2214.4455
2953.7545
3073.0268
3139.3384
3409.2837

TS11+

C	-0.801428000000	0.458369000000	0.169016000000
C	-0.129492000000	-0.814476000000	0.009830000000
H	0.128046000000	1.295320000000	0.271131000000
H	-1.491961000000	0.961746000000	-0.510996000000
N	0.828678000000	0.325651000000	-0.206968000000
H	1.443227000000	0.382479000000	0.622622000000
H	-0.326787000000	-1.362045000000	-0.906516000000
H	0.032247000000	-1.420409000000	0.899464000000

Wavenumbers / cm^{-1}:

-1288.1733
446.1482
692.9722
795.2023
839.8906
993.9972
1074.0665
1148.6897
1176.1579
1220.5174
1287.9677
1495.3764
1523.8778
2287.555
3097.8266
3127.3221
3200.0604
3344.3609

TS12

C	0.121618000000	0.824478000000	-0.191928000000
C	0.901751000000	-0.338744000000	-0.016879000000
H	-1.082007000000	-0.635489000000	0.962008000000
H	0.137431000000	1.585309000000	0.583821000000
N	-0.882057000000	-0.246608000000	0.048679000000
H	-1.557949000000	-0.497489000000	-0.665306000000
H	1.512310000000	-0.597495000000	0.868525000000
H	1.024406000000	-1.042987000000	-0.836959000000

Wavenumbers / cm^{-1}:

-884.8771

472.8813

678.3639

796.0874

877.3936

978.2477

1032.3315

1093.3141

1117.0726

1265.9757

1310.7606

1527.784

1647.58

2904.6767

3102.9518

3125.6061

3452.8499

3535.9106

TS12+

C	0.068491000000	0.722753000000	-0.218591000000
C	1.000543000000	-0.291304000000	0.029474000000
H	-1.444256000000	-0.213118000000	0.935470000000
H	0.011762000000	1.738262000000	0.147267000000
N	-0.919738000000	-0.261611000000	0.065835000000
H	-1.299169000000	-0.860437000000	-0.665279000000
H	1.690369000000	-0.261886000000	0.877647000000
H	1.065258000000	-1.160236000000	-0.621248000000

Wavenumbers / cm^{-1}:

-867.9845
539.8897
611.6955
663.9886
769.9874
940.6487
1064.9191
1083.6696
1133.9421
1249.4694
1347.1796
1485.979
1604.9551
3071.0395
3185.4996
3233.2715
3465.8835
3588.27

HL1

C	1.112338000000	0.145497000000	-0.000003000000
N	-0.219357000000	-0.461864000000	-0.000028000000
C	-1.315182000000	0.065644000000	0.000026000000
H	1.655075000000	-0.199045000000	0.882418000000
H	1.069568000000	1.241507000000	-0.000661000000
H	1.655647000000	-0.200123000000	-0.881640000000
H	-1.627725000000	1.123866000000	-0.000065000000

Wavenumbers / cm^{-1}:

169.9611
341.4712
705.468
895.0119
982.3118
1109.8878
1116.824
1426.6056
1480.3178
1490.7315
1913.2802
2911.4386
2997.9729
3088.1524
3107.4781

HL1+

C	1.223932000000	-0.000047000000	0.000011000000
N	-0.213284000000	0.000045000000	-0.000005000000
C	-1.349923000000	0.000149000000	-0.000002000000
H	1.558477000000	-0.862615000000	0.577899000000
H	1.558499000000	0.931728000000	0.458004000000
H	1.558441000000	-0.069279000000	-1.035954000000
H	-2.426488000000	-0.000765000000	0.000028000000

Wavenumbers / cm^{-1}:

341.9673

341.983

786.0226

786.0301

859.1793

1137.9417

1137.9833

1433.1239

1458.7138

1458.7227

2338.4746

3055.4664

3152.76

3152.8281

3379.0076

HL2

C	0.470788000000	0.706262000000	-0.143230000000
C	-0.832213000000	0.056423000000	0.040638000000
H	1.031107000000	1.353404000000	0.522826000000
N	0.449512000000	-0.687631000000	-0.122281000000
H	0.813004000000	-1.139037000000	0.715353000000
H	-1.521800000000	-0.048700000000	-0.790331000000
H	-1.300343000000	0.071635000000	1.023673000000

Wavenumbers / cm^{-1}:

735.3677

788.8287

881.3353

906.332

981.0839

1066.5582

1094.6393

1186.9865

1221.5514

1337.9743

1515.643

3076.9285

3152.5289

3166.2837

3450.7925

HL2+

C	-0.442165000000	0.663558000000	-0.000011000000
C	0.894467000000	0.015924000000	-0.000003000000
H	-1.102401000000	1.524165000000	0.000022000000
N	-0.486393000000	-0.583001000000	0.000030000000
H	-1.091164000000	-1.406208000000	-0.000111000000
H	1.442276000000	-0.056933000000	0.932657000000
H	1.442228000000	-0.056908000000	-0.932695000000

Wavenumbers / cm^{-1}:

540.9039

694.1764

937.4102

943.2545

1050.1275

1101.346

1127.2668

1139.616

1238.3204

1505.4749

1770.6799

3126.068

3225.0667

3245.7428

3499.6334

HL3

C	1.062119000000	-0.593816000000	0.000000000000
C	0.000000000000	0.451391000000	0.000000000000
H	1.700010000000	-0.472164000000	0.879390000000
H	1.700010000000	-0.472164000000	-0.879390000000
H	0.628473000000	-1.601172000000	0.000000000000
N	-1.229756000000	0.318548000000	0.000000000000
H	-1.792918000000	1.170215000000	0.000000000000

Wavenumbers / cm^{-1}:

154.3055
434.3934
671.6479
899.069
973.725
1041.3334
1170.248
1380.9162
1462.4406
1466.8222
1827.7302
3000.4564
3083.2064
3096.0011
3434.198

HL3+

C	-0.000611000000	-1.268558000000	0.000000000000
C	0.000000000000	0.167785000000	0.000000000000
H	0.516736000000	-1.626708000000	0.896105000000
H	0.516736000000	-1.626708000000	-0.896105000000
H	-1.035500000000	-1.626074000000	0.000000000000
N	0.000689000000	1.309111000000	0.000000000000
H	0.000872000000	2.320344000000	0.000000000000

Wavenumbers / cm^{-1}:

397.9701

398.0025

585.8269

585.8589

917.8649

1039.3156

1039.3759

1388.4616

1425.6813

1425.7524

2381.8717

3025.5032

3107.253

3107.6031

3678.9645

HL4

C	-1.278014000000	0.106120000000	-0.015822000000
C	-0.064383000000	-0.413692000000	0.037387000000
H	-2.150694000000	-0.476413000000	0.254194000000
H	-1.448975000000	1.126382000000	-0.365908000000
N	1.181352000000	0.123903000000	-0.048397000000
H	1.936172000000	-0.488959000000	-0.318309000000
H	1.448416000000	0.817102000000	0.639411000000

Wavenumbers / cm^{-1}:

240.1303
383.0075
489.8255
670.4152
834.2156
983.9061
1081.3342
1189.2429
1420.7268
1640.2158
1711.758
3056.3267
3187.5105
3529.974
3634.4743

HL4+

C	-1.326087000000	-0.000190000000	-0.000008000000
C	-0.044778000000	0.000625000000	0.000001000000
H	-1.877473000000	0.938255000000	0.003598000000
H	-1.876380000000	-0.939275000000	-0.003563000000
N	1.212078000000	-0.000148000000	0.000001000000
H	1.747245000000	-0.004138000000	0.871192000000
H	1.747248000000	0.003579000000	-0.871191000000

Wavenumbers / cm^{-1}:

348.0952

393.9454

814.5319

875.2995

914.8133

936.0925

1128.8325

1156.5336

1383.5097

1638.4366

2118.1731

3112.1395

3198.2041

3422.6797

3513.0814

HL5

C	-1.200490000000	-0.167740000000	0.000021000000
C	0.072246000000	0.409544000000	-0.000009000000
H	-2.096893000000	0.439198000000	0.000035000000
H	-1.292376000000	-1.247142000000	0.000037000000
N	1.150066000000	-0.342279000000	-0.000020000000
H	0.123662000000	1.504736000000	0.000021000000
H	1.984605000000	0.248337000000	-0.000025000000

Wavenumbers / cm^{-1}:

491.001

533.8663

672.3127

819.4293

996.1458

1049.7798

1110.2428

1249.5115

1351.5397

1457.8451

1512.6149

3028.1458

3145.4014

3253.414

3442.5863

HL5+

C	1.170877000000	-0.181209000000	-0.000084000000
C	-0.087856000000	0.470906000000	0.000029000000
H	2.112224000000	0.367264000000	-0.000025000000
H	1.185548000000	-1.270465000000	0.000038000000
N	-1.090344000000	-0.358873000000	0.000031000000
H	-0.159972000000	1.558315000000	0.000067000000
H	-2.003523000000	0.118818000000	0.000033000000

Wavenumbers / cm^{-1}:

225.8718
496.6493
750.9009
1002.4544
1095.901
1118.4235
1214.2438
1277.2782
1393.3782
1496.4679
1553.4366
3114.9928
3137.664
3234.6477
3400.0195

HL6

C	-1.175135000000	-0.194928000000	-0.000038000000
C	0.070568000000	0.440571000000	0.000009000000
H	-2.097321000000	0.373422000000	-0.000097000000
H	-1.246604000000	-1.278086000000	-0.000055000000
N	1.251830000000	-0.135267000000	0.000054000000
H	0.078114000000	1.532494000000	-0.000101000000
H	1.130400000000	-1.154818000000	0.000045000000

Wavenumbers / cm^{-1}:

493.7773
493.9358
681.6681
826.7361
987.6697
1078.4053
1140.1593
1239.2749
1378.9757
1452.2914
1497.65
3091.4345
3129.2297
3232.4318
3387.7308

HL6+

C	1.155033000000	-0.204043000000	-0.000365000000
C	-0.080703000000	0.482273000000	-0.000166000000
H	2.102556000000	0.335151000000	0.001565000000
H	1.195036000000	-1.293755000000	0.000416000000
N	-1.218494000000	-0.156921000000	0.000161000000
H	-0.103420000000	1.569708000000	0.000114000000
H	-1.110695000000	-1.182034000000	-0.000037000000

Wavenumbers / cm^{-1}:

-162.4363

491.189

635.4787

988.1747

1094.1552

1141.16

1197.541

1277.4182

1407.1259

1502.0549

1546.4163

3106.3461

3167.1873

3217.1726

3388.7152

HL7

C	0.834374000000	-0.781461000000	0.000000000000
C	0.000000000000	0.483224000000	0.000000000000
H	1.478331000000	-0.800921000000	0.883613000000
H	1.478331000000	-0.800921000000	-0.883613000000
H	0.194145000000	-1.663037000000	0.000000000000
N	-1.244123000000	0.516589000000	0.000000000000
H	0.551807000000	1.438180000000	0.000000000000

Wavenumbers / cm^{-1}:

188.5822

430.76

745.4589

905.4718

1047.03

1066.0197

1245.5509

1385.1212

1475.2694

1480.9893

1731.3603

2948.283

3033.2146

3093.0779

3134.6978

HL7+

6	-0.000073000	-1.223697000	0.000000000
6	0.000048000	1.349851000	0.000000000
1	0.518999000	-1.558565000	0.899111000
1	0.518999000	-1.558565000	-0.899111000
1	-1.038277000	-1.558518000	0.000000000
7	0.000000000	0.213190000	0.000000000
1	0.000431000	2.426397000	0.000000000

Wavenumbers / cm^{-1}:

342.0812
342.0894
785.8672
785.8677
860.0908
1137.9377
1137.95
1433.2923
1458.734
1458.742
2338.4614
3055.2454
3152.4143
3152.4218
3379.155

HL8

C	-0.000012000000	-0.155860000000	1.138802000000
C	-0.000012000000	-0.155860000000	-1.138802000000
H	0.000067000000	0.401325000000	2.069067000000
H	0.000048000000	-1.250933000000	1.182515000000
N	-0.000012000000	0.509934000000	0.000000000000
H	0.000048000000	-1.250933000000	-1.182515000000
H	0.000067000000	0.401325000000	-2.069067000000

Wavenumbers / cm^{-1}:

340.8721

472.3971

588.6043

849.6064

871.3304

1105.7659

1172.8621

1278.2002

1295.2855

1493.9096

1546.8445

3011.8473

3024.8492

3186.5515

3189.1483

HL8+

C	0.000001000000	0.167233000000	1.113695000000
C	0.000001000000	0.167233000000	-1.113695000000
H	-0.000021000000	-0.370621000000	2.061466000000
H	0.000014000000	1.264671000000	1.129096000000
N	0.000001000000	-0.542128000000	0.000000000000
H	0.000014000000	1.264671000000	-1.129096000000
H	-0.000021000000	-0.370621000000	-2.061466000000

Wavenumbers / cm^{-1}:

-778.9126
356.0597
488.0981
1129.7597
1183.2739
1223.0597
1231.2931
1311.1906
1469.8537
1574.8321
1590.8274
3036.7342
3039.687
3191.3241
3191.9274

H L9

6	1.138823000	0.155849000	0.000019000
7	-0.000050000	-0.509913000	-0.000059000
6	-1.138757000	0.155843000	-0.000044000
1	2.069062000	-0.401374000	0.000080000
1	1.182572000	1.250919000	0.000034000
1	-1.182577000	1.250936000	0.000318000
1	-2.069101000	-0.401243000	0.000128000

Wavenumbers / cm^{-1}:

340.8361
472.3748
588.6214
849.6787
871.3901
1105.7995
1172.9021
1278.2227
1295.4211
1493.9594
1546.9058
3011.7487
3024.7566
3186.4555
3189.0678

HL9+

C	-1.247142000000	0.000002000000	-0.000001000000
N	-0.000001000000	-0.000002000000	0.000002000000
C	1.247150000000	0.000000000000	-0.000002000000
H	-1.785375000000	-0.037380000000	0.947061000000
H	-1.785377000000	0.037374000000	-0.947061000000
H	1.785359000000	-0.947071000000	-0.037374000000
H	1.785348000000	0.947077000000	0.037378000000

Wavenumbers / cm^{-1}:

340.5665

340.572

909.2232

1071.728

1071.7499

1128.1529

1128.1649

1193.4546

1453.9507

1517.713

2091.0925

3078.7083

3093.0945

3196.3067

3196.3548

HL10

C	1.055028000000	-0.589298000000	0.000000000000
C	0.000000000000	0.461611000000	0.000000000000
H	1.693572000000	-0.476099000000	0.879491000000
H	1.693572000000	-0.476099000000	-0.879491000000
H	0.620329000000	-1.599806000000	0.000000000000
N	-1.223248000000	0.524015000000	0.000000000000
H	-1.774911000000	-0.349977000000	0.000000000000

Wavenumbers / cm^{-1}:

153.966

405.9255

645.4166

893.0926

929.9322

1037.5473

1050.4872

1382.3205

1458.2963

1467.3355

1903.8584

2970.3413

3076.6944

3101.8214

3206.7744

HL10+

C	-0.000611000000	-1.268558000000	0.000000000000
C	0.000000000000	0.167785000000	0.000000000000
H	0.516736000000	-1.626708000000	0.896105000000
H	0.516736000000	-1.626708000000	-0.896105000000
H	-1.035500000000	-1.626074000000	0.000000000000
N	0.000689000000	1.309111000000	0.000000000000
H	0.000872000000	2.320344000000	0.000000000000

Wavenumbers / cm^{-1}:

397.9701

398.0025

585.8269

585.8589

917.8649

1039.3156

1039.3759

1388.4616

1425.6813

1425.7524

2381.8717

3025.5032

3107.253

3107.6031

3678.9645

E-HCNH

N	0.556879000000	-0.174842000000	-0.000001000000
C	-0.618077000000	0.195188000000	0.000001000000
H	-1.482921000000	-0.481661000000	0.000000000000
H	1.293235000000	0.534426000000	0.000000000000

Wavenumbers / cm^{-1}:

913.9833

973.8539

1199.84

1802.9823

2996.0282

3417.6581

Z-HCNH

N	0.586356000000	-0.100457000000	-0.000005000000
H	1.114079000000	0.782527000000	-0.000010000000
C	-0.633914000000	-0.121981000000	0.000006000000
H	-1.415082000000	0.652558000000	0.000012000000

Wavenumbers / cm^{-1}:

874.9734

904.6808

1001.2685

1861.9223

2948.3777

3281.0692

$HCNH^+$

C	0.008041000000	0.599459000000	0.000000000000
N	0.008041000000	-0.532624000000	0.000000000000
H	-0.037603000000	1.677855000000	0.000000000000
H	-0.066927000000	-1.546242000000	0.000000000000

Wavenumbers / cm^{-1}:

668.1894

762.8872

837.4285

2247.1635

3334.9216

3643.1845

NCH2

C	0.000000000000	0.000000000000	-0.502364000000
N	0.000000000000	0.000000000000	0.738868000000
H	0.000000000000	0.936600000000	-1.078945000000
H	0.000000000000	-0.936600000000	-1.078945000000

Wavenumbers / cm^{-1}:

932.5514

988.4808

1378.1795

1720.5664

2955.922

3008.9135

H_2NC

N	0.000000000000	0.000000000000	0.442408000000
H	0.000000000000	0.858948000000	1.001586000000
C	0.000000000000	0.000000000000	-0.850005000000
H	0.000000000000	-0.858948000000	1.001586000000

Wavenumbers / cm^{-1}:

732.0216

1029.5073

1438.343

1624.0809

3309.8377

3346.7154

Vinyl

C	0.048317000000	-0.585305000000	0.000000000000
H	0.968698000000	-1.165854000000	0.000000000000
H	-0.879712000000	-1.164394000000	0.000000000000
C	0.048317000000	0.719164000000	0.000000000000
H	-0.668789000000	1.527093000000	0.000000000000

Wavenumbers / cm^{-1}:

711.9455

819.504

921.8812

1046.1633

1391.4347

1650.4727

3036.4381

3132.9776

3234.0562

CH_3

C	0.000000000000	0.000000000000	0.000000000000
H	0.000000000000	1.080614000000	0.000000000000
H	-0.935839000000	-0.540307000000	0.000000000000
H	0.935839000000	-0.540307000000	0.000000000000

Wavenumbers / cm^{-1}:

506.4384

1403.3597

1403.3598

3102.2638

3281.1941

3281.1944

CH_3^+

C	0.000000000000	0.000000000000	0.000000000000
H	0.000000000000	1.093336000000	0.000000000000
H	-0.946857000000	-0.546668000000	0.000000000000
H	0.946857000000	-0.546668000000	0.000000000000

Wavenumbers / cm-1:

1406.0702

1406.0702

1417.9386

3020.9002

3216.507

3216.5071

N H 2

N	0.000000000000	0.000000000000	0.143964000000
H	0.000000000000	0.801927000000	-0.503875000000
H	0.000000000000	-0.801927000000	-0.503875000000

Wavenumbers / cm-1:

1535.6349

3332.579

3417.7981
